# Circular stapler platform and the risk of anastomotic leakage after elective left colectomy and high anterior resection

**DOI:** 10.1111/codi.70564

**Published:** 2026-08-03

**Authors:** Stefano Guadagni, Marco Catarci, Francesco Masedu, the Italian ColoRectal Anastomotic Leakage (iCral) study group, Marco Clementi, Paolo Ciano, Michele Benedetti, Leonardo Antonio Montemurro, Fanny Massimi, Elena Belloni, Matteo Di Carlo, Giacomo Ruffo, Elisa Bertocchi, Gaia Masini, Massimo Giuseppe Viola, Amedeo Altamura, Francesco Rubichi, Gianluca Garulli, Daniele Parlanti, Gabriele Vago, Carlo Di Marco, Marco Scatizzi, Lorenzo Pandolfini, Alessandro Falsetto, Vincenzo Bottino, Antonio Ferronetti, Ferdinando Ficari, Francesco Giudici, Fabio Cianchi, Paolo Delrio, Massimiliano Di Marzo, Felice Pirozzi, Antonio Sciuto, Maria Carmela Giuffrida, Marco Migliore, Raffaele De Luca, Michele Simone, Alberto Patriti, Valerio Sisti, Marcella Lodovica Ricci, Walter Siquini, Alessandro Cardinali, Stefano D'Ugo, Marcello Spampinato, Stefano Scabini, Alessandra Aprile, Domenico Soriero, Marco Caricato, Gabriella Teresa Capolupo, Giusto Pignata, Jacopo Andreuccetti, Ilaria Canfora, Andrea Liveran, Giuseppe Lamacchia, Claudia Franceschilli, Roberto Campagnacci, Angela Maurizi, Pierluigi Marini, Grazia Maria Attinà, Ugo Elmore, Lorenzo Gozzini, Francesco Corcione, Umberto Bracale, Roberto Peltrini, Maria Michela Di Nuzzo, Roberto Santoro, Pietro Amodio, Massimo Carlini, Domenico Spoletini, Rosa Marcellinaro, Antonio Giuliani, Giovanni Del Vecchio, Mario Sorrentino, Massimo Stefanoni, Giovanni Ferrari, Carmelo Magistro, Diletta Cassini, Alberto Di Leo, Lorenzo Crepaz, Augusto Verzelli, Andrea Budassi, Giuseppe Sica, Giulia Bagaglini, Stefano Rausei, Silvia Tenconi, Davide Cavaliere, Leonardo Solaini, Giorgio Ercolani, Sarah Molfino, Marco Milone, Giovanni Domenico De Palma, Giovanni Ciaccio, Paolo Locurto, Giovanni Tebala, Antonio Di Cintio, Luigi Boni, Elisa Cassinotti, Stefano Mancini, Andrea Sagnotta, Mario Guerrieri, Monica Ortenzi, Roberto Persiani, Alberto Biondi, Andrea Lucchi, Giulia Vitali, Dario Parini, Maurizio De Luca, Antonino Spinelli, Francesco Carrano, Michele Genna, Francesca Fior, Andrea Coratti, Giuseppe Giuliani, Dario Scala, Graziella Marino, Andrea Muratore, Patrizia Marsanic, Umberto Rivolta, Giuliano Sarro, Micaela Piccoli, Francesca Pecchini, Carlo Talarico, Vincenzo Greco, Michele Motter, Giuseppe Tirone, Mauro Totis, Nicolò Tamini, Marco Braga, Franco Roviello, Roberto Piagnerelli, Alessandro Anastasi, Giuseppe Canonico, Gianluca Guercioni, Simone Cicconi, Irene Marziali, Giuseppe Maria Ettorre, Marco Colasanti, Mauro Montuori, Enrico Pinotti, Pierpaolo Mariani, Roberta Carminati, Nicolò de Manzini, Edoardo Osenda, Nicolò de Manzini, Annibale Donini, Luigina Graziosi, Mariano Fortunato Armellino, Ciro De Martino, Lucio Taglietti, Silvia Ruggiero, Gabriele Anania, Matteo Chiozza, Mariantonietta Di Cosmo, Daniele Zigiotto, Carlo Vittorio Feo, Fioralba Pindozzi, Paolo Millo, Manuela Grivon, Corrado Pedrazzani, Cristian Conti, Silvio Guerriero, Lorenzo Organetti, Andrea Costanzi, Michela Monteleone, Nereo Vettoretto, Emanuele Botteri, Federico Marchesi, Giorgio Dal Monte, Massimo Basti, Diletta Frazzini, Graziano Longo, Simone Santoni, Moreno Cicetti, Gabriele La Gioia, Giuseppe Brisinda, Maria Michela Chiarello, Maria Cariati, Stefano Berti, Andrea Gennai, Ugo Pace, Silvia De Franciscis, Felice Borghi, Luca Pellegrino, Gianandrea Baldazzi, Gian Luca Baiocchi, Agata Borsella, Andrea Gattolin, Valentina Testa, Michele Masetti, Chiara Giordano, Leonardo Vincenti, Valeria Andriola, Massimiliano Fabozzi, Giovanna Ioia, Francesco Stanzione, Domenico Manzolillo, Gianluigi Moretto, Harmony Impellizzeri, Isacco Montroni, Giacomo Frascaroli, Massimiliano Di Paola, Alessandro Arturi, Luca Morelli, Gregorio Di Franco, Fausto Catena, Nicola Zanini, Salvatore Ramuscello, Silvia Mancini, Alessandro Carrara, Marinella Menegazzo

**Affiliations:** ^1^ General Surgery Unit and Department of Biotechnological and Applied Clinical Sciences Università Degli Studi Dell'aquila L'Aquila Italy; ^2^ General Surgery Unit Sandro Pertini Hospital Rome Italy; ^3^ Department of Biotechnological and Applied Clinical Sciences Università Degli Studi Dell'aquila L'Aquila Italy

**Keywords:** anastomotic leakage, circular stapler, colorectal surgery, comparative effectiveness, generalized boosted mode, propensity score

## Abstract

**Aim:**

The impact of different circular stapler platforms on the risk of anastomotic leakage following elective left colectomy and high anterior resection remains poorly defined. We compared the risk of anastomotic leakage across commonly used circular stapler platforms using advanced confounding control methods.

**Methods:**

This pre‐planned post hoc analysis pooled individual patient data from the published iCral‐2 and iCral‐3 studies and from the iCral‐4 study, whose primary results are currently under peer review. Adults undergoing elective left colectomy or high anterior resection with standardized end‐to‐end circular stapled anastomosis were included. Five circular stapler platforms were compared. Confounding was addressed using generalized boosted model–based propensity score weighting targeting the average treatment effect on the treated, with doubly robust outcome modelling. The primary endpoint was overall anastomotic leakage at 60 days.

**Results:**

Among 3,653 patients, overall anastomotic leakage occurred in 2.7% of patients receiving the reference 3‐row platform. After weighting, Platforms 3 and 4 were associated with a significantly increased risk of overall anastomotic leakage (odds ratios >2), whereas no statistically significant differences were observed for Platforms 2 or 5. Because the effective sample size after weighting was substantially reduced for Platform 3 owing to limited covariate overlap, the corresponding effect estimate should be interpreted with appropriate caution.

**Conclusions:**

Selected circular stapler platforms were associated with an increased risk of overall anastomotic leakage. These findings suggest platform‐specific rather than class‐wide differences and warrant confirmation in prospective studies designed to identify the device‐related and procedural factors underlying these associations.

**Trial Registration:**

NCT03771456 for iCral2, NCT04397627 for iCral3 and NCT05227014 for iCral4.

**What does this paper add to the literature?**

Using pooled multicentre data and advanced propensity‐based methods, we compared anastomotic outcomes across five circular stapler platforms in elective left colectomy and high anterior resection. Selected stapler platforms were associated with an increased risk of anastomotic leakage compared with the reference platform, suggesting platform‐specific rather than class‐wide differences.

## INTRODUCTION

Anastomotic leakage (AL) after left colectomy and anterior resection remains one of the most severe complications in colorectal surgery, with substantial clinical, oncological and economic consequences [1].

Risk factors for AL include patient‐, disease‐ and technical‐related determinants [2]. Among these, technical factors are particularly attractive because they are potentially modifiable.

Within this context, the circular stapler platform represents a potentially modifiable technical determinant of anastomotic integrity. Since the introduction of mechanical circular staplers [3], multiple platforms with distinct design features have been developed, each intended to improve anastomotic integrity. However, whether these design differences translate into clinically meaningful differences in anastomotic outcomes remains controversial.

The impact of circular stapler platforms on AL has been explored mainly through retrospective observational studies, including propensity score–matched analyses [4, 5, 6, 7, 8, 9, 10, 11, 12, 13] and meta‐analyses [14, 15, 16, 17, 18]. While meta‐analyses synthesize available evidence, their conclusions remain influenced by differences in study selection, device classification, comparator definitions and statistical methodology. Randomized controlled trials comparing multiple stapler platforms are unlikely to be feasible because of limited equipoise, device heterogeneity and logistical constraints. Consequently, propensity score–based methods have become increasingly common in comparative effectiveness research. However, conventional pairwise matching is suboptimal for multiple treatment comparisons because it may produce heterogeneous reference groups, reduce sample size and limit generalizability. Comparisons across studies are further complicated by methodological differences, including target estimands (average treatment effect vs. average treatment effect on the treated), covariate selection, matching algorithms, calliper width, sample size and eligibility criteria.

Accordingly, comparative analyses that address real‐world heterogeneity while maintaining methodological rigour are needed. The present study addresses this gap by comparing multiple circular stapler platforms in a large multicentre cohort using an advanced multi‐treatment propensity‐based analytical framework.

## METHODS

### Study design

This pre‐planned post hoc retrospective analysis used a prospectively collected multicentre database including patients who underwent colorectal resection with anastomosis for malignant or benign disease. Individual patient‐level data were pooled from three consecutive studies of the Italian ColoRectal Anastomotic Leakage (iCral) study group (iCral‐2, iCral‐3 and iCral‐4), all of which predefined anastomotic leakage as a study endpoint. The aim was to evaluate the association between circular stapler platform and the risk of anastomotic leakage using advanced confounding control methods.

Data from the published iCral‐2 [19] and iCral‐3 [20] studies and from the iCral‐4 study, whose primary results are currently under peer review, were combined to address a research question that was not specified as a primary or secondary endpoint in the original study protocols.

Eligibility criteria for the three‐iCral studies are summarized in the Supporting Information. Differences among studies in American Society of Anesthesiologists (ASA) class distribution, urgent surgery and protective stoma use were harmonized in the present analysis.

To facilitate assessment of the pooled dataset, the Supporting Information includes descriptive characteristics of the three constituent cohorts (Table S1) and a sensitivity analysis restricted to the published iCral‐2/iCral‐3 cohorts (Table S2).

### Study population

Among the 11,849 patients in the overall cohort, 7,394 (62.4%) underwent circular stapled anastomosis. Both malignant and benign indications were included because the stapled end‐to‐end anastomotic technique was considered technically comparable despite differences in resection extent and vascular control. Potential confounding by underlying pathology was addressed by including surgery for malignancy as a balancing covariate in the Generalized Boosted Models (GBM).

To minimize procedural and anatomical confounding and to ensure a technically homogeneous comparison across circular stapler platforms—prioritizing internal over external validity—additional exclusion criteria were applied. Patients with incomplete data, urgent surgery, ASA class IV–V or protective stoma proximal to the anastomosis were excluded. Procedures with substantially different surgical anatomy or anastomotic configuration (i.e. resections other than left colectomy or anterior resection and anastomoses other than end‐to‐end) were also excluded. Conditions known to exert a disproportionate subgroup‐specific effect on anastomotic leakage, namely anastomotic height <5 cm from the external anal verge and receipt of neoadjuvant therapy, were excluded. Circular staplers with a 25‐mm diameter were excluded because this size was unavailable across all compared platforms.

**FIGURE 1:**
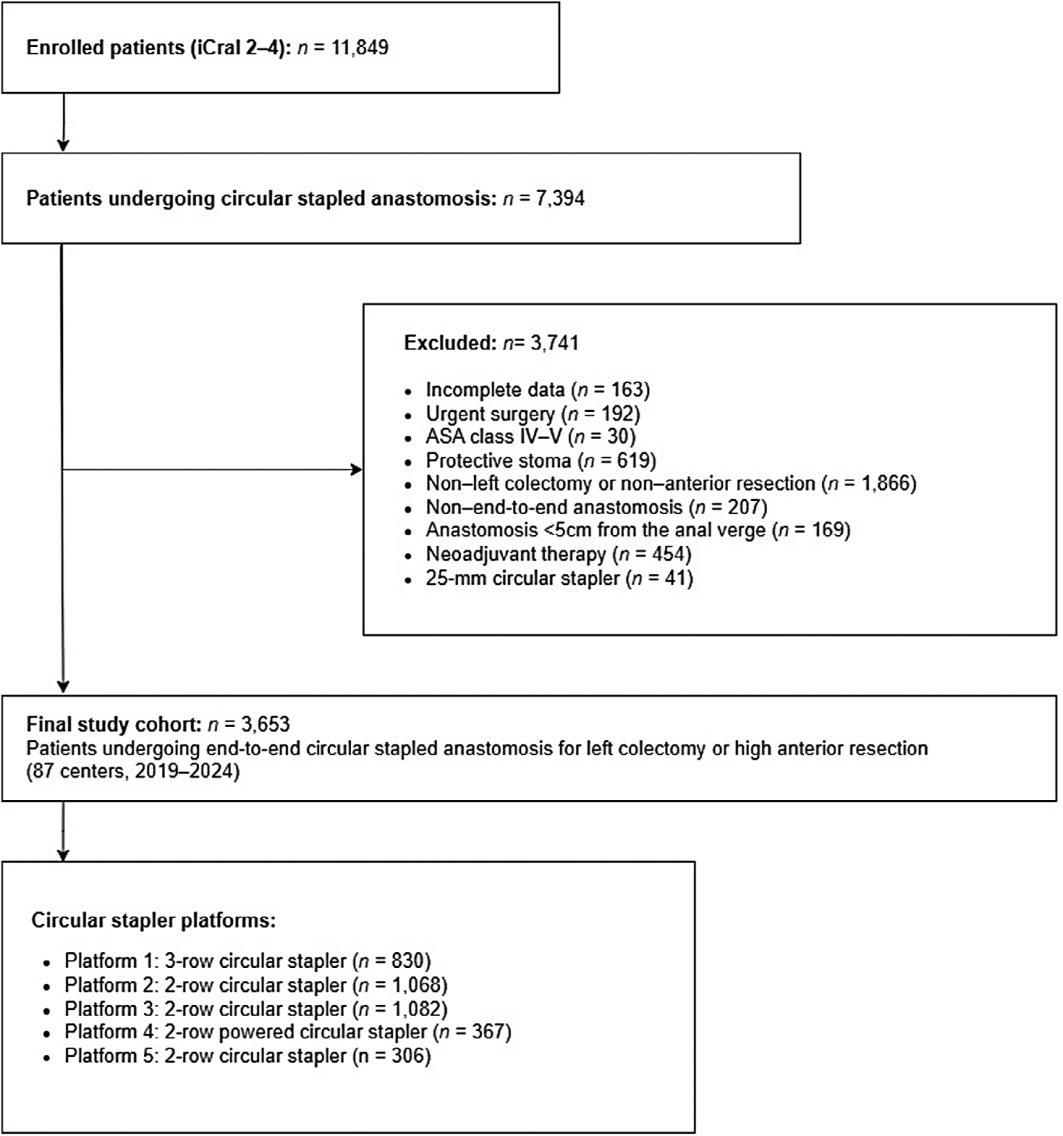
Flow diagram of patient selection from the pooled iCral 2–4 cohorts. Of 11,849 enrolled patients, 7,394 underwent circular stapled anastomosis. After application of predefined exclusion criteria, 3,653 patients undergoing standardized end‐to‐end circular stapled anastomosis for left colectomy or high anterior resection comprised the final study cohort.

After application of all exclusion criteria, 3,653 patients undergoing standardized end‐to‐end circular stapled anastomosis for left colectomy (i.e. anastomosis >18 cm from the external anal verge) or high anterior resection (i.e. anastomosis 5–18 cm from the external anal verge) constituted the final study population. When a circular stapler was used, manufacturer, model and diameter were prospectively recorded, allowing precise platform classification. Stapler selection was neither randomized nor protocolized; therefore, multivariable adjustment and propensity‐based methods were applied to mitigate confounding related to patient‐, procedure‐, and centre‐level characteristics. Patients were categorized into five circular stapler platforms based on manufacturer‐specific groupings encompassing devices with shared staple‐line architecture and mechanical design: Platform 1 (3‐row circular stapler, *n* = 830), Platform 2 (2‐row circular stapler, *n* = 1,068), Platform 3 (2‐row circular stapler, *n* = 1,082), Platform 4 (2‐row powered circular stapler, *n* = 367) and Platform 5 (2‐row circular stapler, *n* = 306). The study flow diagram is shown in Figure 1.

### Participating centres

A total of 3,653 patients were enrolled across 87 Italian surgical centres between January 2019 and January 2024. The median enrolment was 19 patients per centre; six centres enrolled only one patient, 27 enrolled fewer than 10 and 39 enrolled fewer than 20. Five circular stapler platforms were used across the study population, although platform availability and preference varied by centre. Six centres used a single platform, whereas five used all five platforms.

### Descriptive variables

**FIGURE 2:**
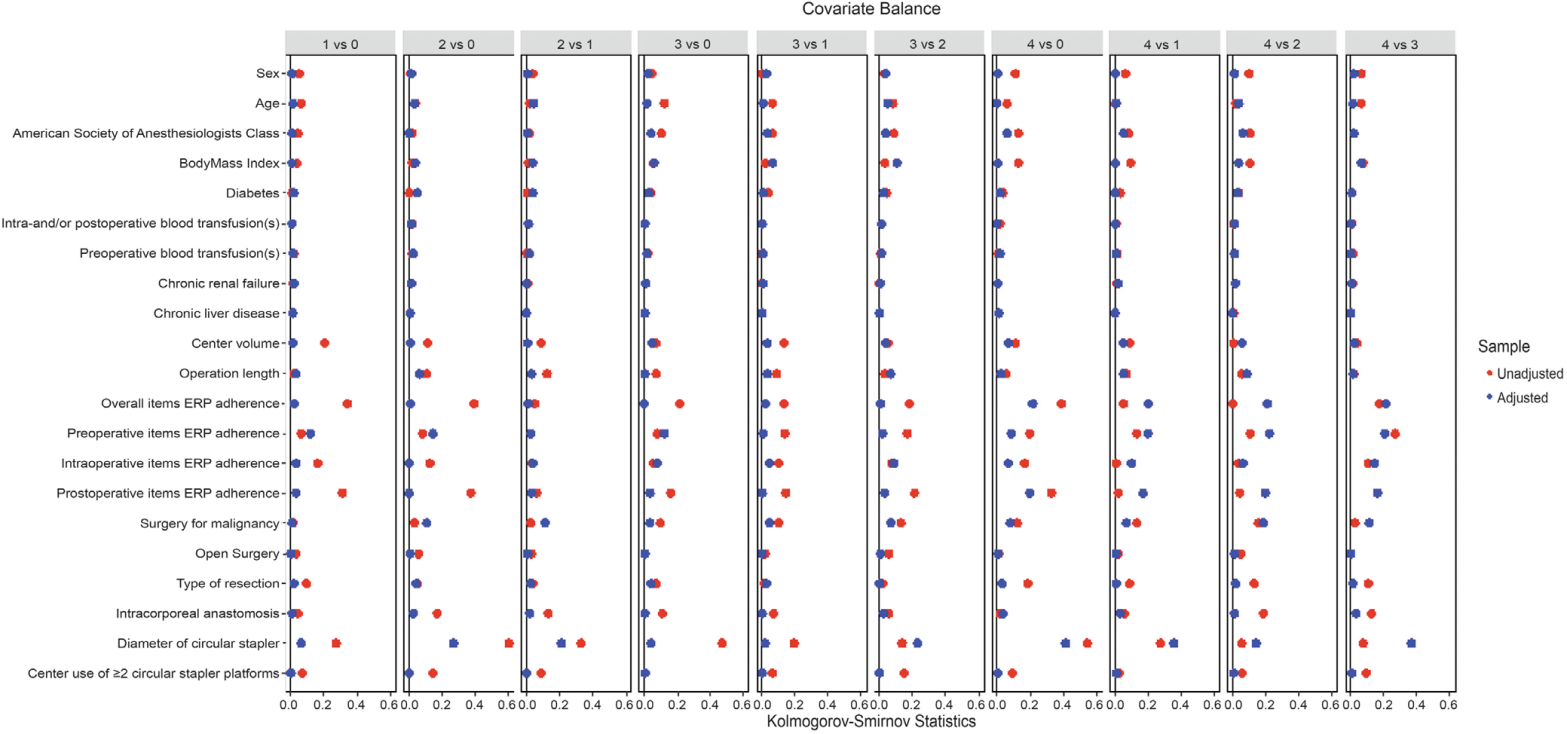
Love plot of covariate balance before and after adjustment using generalized boosted models. Love plot displaying Kolmogorov–Smirnov statistics for baseline covariates across pairwise comparisons of the five circular stapler platforms included in the study. Platform 1 (3‐row circular stapler), labelled as group “0” in the figure, served as the reference category and was compared with Platform 2 (2‐row circular stapler, “1”), Platform 3 (2‐row circular stapler, “2”), Platform 4 (2‐row powered circular stapler, “3”) and Platform 5 (2‐row circular stapler, “4”). Red dots indicate unadjusted comparisons, whereas blue dots indicate comparisons after generalized boosted model weighting. Improved covariate balance after adjustment is reflected by lower Kolmogorov–Smirnov statistics. ERP, Enhanced Recovery Pathway.

Baseline characteristics stratified by circular stapler platform are reported in Table 1. Twenty‐one descriptive variables are summarized in Figure 2. Continuous variables were dichotomized at their median values. Centres were classified as high‐volume (≥4 cases/month) or low‐volume (<4 cases/month) based on the median monthly enrolment. Each centre was additionally characterized by a binary variable indicating use of ≥2 circular stapler platforms. Adherence to ERP protocols was evaluated using 20 items common to all three iCral study protocols, encompassing the preoperative, intraoperative and postoperative phases (Table S3). Open surgery was included as an individual covariate because of its established clinical relevance. The complementary variable, minimally invasive surgery, also contributed to the composite intraoperative ERP adherence score, which was incorporated as a summary measure of overall adherence to the perioperative care pathway.

**TABLE 1:** Baseline characteristics of the study population according to circular stapler platform.

Descriptive variables		Overall patients		Platform 1 (3‐row CS)		Platform 2 (2‐row CS)		Platform 3 (2‐row CS)		Platform 4 (2‐row powered CS)		Platform 5 (2‐row CS)		^a^ p
		No. = 3,653		No. = 830		No. = 1,068		No. = 1,082		No. = 367		No. = 306		
	Pattern	No.	%	No.	%	No.	%	No.	%	No.	%	No.	%	
**Patient‐related**														
Age (years)	< 64.75	1,832	50.2	377	45.4	550	51.5	536	49.5	212	57.8	157	51.3	0.002
	≥ 64.75	1,821	49.8	453	54.6	518	48.5	546	50.5	155	42.2	149	48.7	
Gender	Male	1,750	47.9	424	51.1	493	46.2	540	49.9	171	46.6	122	39.9	0.006
	Female	1,903	52.1	406	48.9	575	53.8	542	50.1	196	53.4	184	60.1	
ASA class	I‐II	2,762	75.6	595	71.7	810	75.8	797	73.7	302	82.3	258	84.3	0.000
	III	891	24.4	235	28.3	258	24.2	285	26.3	65	17.7	48	15.7	
Body Mass Index (Kg/m^2^)	< 24.93	1,835	50.2	391	47.1	538	50.4	531	49.1	192	52.3	183	59.8	0.003
	≥ 24.93	1,818	49.8	439	52.9	530	49.6	551	50.9	175	47.7	123	40.2	
Diabetes	Yes	373	10.2	91	11.0	113	10.6	121	11.2	25	6.8	23	7.5	0.065
	No	3,280	89.8	739	89.0	955	89.4	961	88.8	342	93.2	283	92.5	
Chronic renal failure	Yes	99	2.7	29	3.5	27	2.5	23	2.1	8	2.2	12	3.9	0.234
	No	3,554	97.3	801	96.5	1,041	97.5	1,059	97.9	359	97.8	294	96.1	
Chronic liver disease	Yes	30	0.8	12	1.4	6	0.6	8	0.7	3	0.8	1	0.3	0.209
	No	3,623	99.2	818	98.6	1,062	99.4	1,074	99.3	364	99.2	305	99.7	
**Centre‐related**														
Centre volume	< 4 cases/month	903	24.7	114	13.7	362	33.9	275	25.4	76	20.7	76	24.8	0.000
	≥ 4 cases/month	2,750	75.3	716	86.3	706	66.1	807	74.6	291	79.3	230	75.2	
Centre use of ≥2 circular stapler platforms	< 2	265	7.3	2	0.2	71	6.7	163	15.1	0	0.0	29	9.5	0.000
	≥ 2	3,388	92.7	828	99.8	997	93.4	919	84.9	367	100	277	90.5	
**Surgical treatment‐related**														
Surgery for malignancy	Yes	2,075	56.8	477	57.5	623	58.3	660	61.0	176	48.0	139	45.4	0.000
	No	1,578	43.2	353	42.5	445	41.7	422	39.0	191	52.0	167	54.6	
	Diverticular disease (*n* = 1,058)													
	Endometriosis (*n* = 365)													
	Polyps (*n* = 106)													
	Inflammatory bowel disease (*n* = 7)													
	Other (*n* = 42)													
Preoperative BT(s)	Yes	63	1.7	26	3.1	13	1.2	13	1.2	3	0.8	8	2.6	0.003
	No	3,590	98.3	804	96.9	1,055	98.8	1,069	98.8	364	99.2	298	97.4	
Intra/postoperative BT(s)	Yes	125	3.4	21	2.5	34	3.2	47	4.3	11	3.0	12	3.9	0.249
	No	3,528	96.6	809	97.5	1,034	96.8	1,035	95.7	356	97.0	294	96.1	
Mini‐invasive surgery	No	163	4.5	15	1.8	48	4.5	84	7.8	8	2.2	8	2.6	0.000
	Yes	3,490	95.5	815	98.2	1,020	95.5	998	92.2	359	97.8	298	97.4	
	Laparoscopic (*n* = 3,039)													
	Robotic (*n* = 328)													
	Converted (*n* = 123)													
Type of resection	Left colectomy	2,528	69.2	628	75.7	712	66.7	761	70.3	251	68.4	176	57.5	0.000
	Anterior resection	1,125	30.8	202	24.3	356	33.3	321	29.7	116	31.6	130	42.5	
Diameter of circular stapler	< 30 mm	2,476	67.8	271	32.7	639	59.8	1,007	93.1	292	79.6	267	87.3	0.000
	≥ 30 mm	1,177	32.2	559	67.3	429	40.2	75	6.9	75	20.4	39	12.7	
Operation length (minutes)	< 180	1,933	52.9	471	56.8	624	58.4	499	46.1	181	49.3	158	51.6	0.000
	≥ 180	1,720	47.1	359	43.2	444	41.6	583	53.9	186	50.7	148	48.4	
Anastomotic site	Intracorporeal	3,170	86.8	779	93.9	960	89.9	834	77.1	304	82.8	293	95.7	0.000
	Extracorporeal	483	13.2	51	6.1	108	10.1	248	22.9	63	17.2	13	4.3	
**ERP programme items adherence**														
Overall items adherence (%)	< 75	1,862	51.0	199	24.0	622	58.2	682	63.0	166	45.2	193	63.1	0.000
	≥ 75	1,791	49.0	631	76.0	446	41.8	400	37.0	201	54.8	113	36.9	
Preoperative items adherence (%)	< 57.14	1,472	40.3	292	35.2	439	41.1	474	43.8	100	27.3	167	54.6	0.000
	≥ 57.14	2,181	59.7	538	64.8	629	58.9	608	56.2	267	72.7	139	45.4	
Intraoperative items adherence (%)	< 88.89	2,886	79.0	570	68.7	901	84.4	882	81.5	272	74.1	261	85.3	0.000
	≥ 88.89	767	21.0	260	31.3	167	15.6	200	18.5	95	25.9	45	14.7	
Postoperative items adherence (%)	< 75	1,905	52.2	230	27.7	626	58.6	703	65.0	161	43.9	185	60.5	0.000
	≥ 75	1,748	47.8	600	72.3	442	41.4	379	35.0	206	56.1	121	35.9	
**60‐day adverse events**														
Overall anastomotic leakage	Yes	176	4.8	22	2.6	50	4.7	73	6.7	19	5.2	12	3.9	0.001
	No	3,477	95.2	808	97.4	1,018	95.3	1,009	93.3	348	94.8	294	96.1	
^b^ Major anastomotic leakage	Yes	143	3.9	20	2.4	41	3.8	59	5.4	15	4.1	8	2.6	0.010
	No	3,510	96.1	810	97.6	1,027	96.2	1,023	94.6	352	95.9	298	97.4	
Overall anastomotic bleeding	Yes	127	3.5	13	1.6	44	4.1	43	4.0	8	2.2	19	6.2	0.001
	No	3,526	96.5	817	98.4	1,024	95.9	1,039	96.0	359	97.8	287	93.8	
^b^ Major anastomotic bleeding	Yes	49	1.3	3	0.4	22	2.1	17	1.6	6	1.6	1	0.3	0.010
	No	3,604	98.7	827	99.6	1,046	97.9	1,065	98.4	361	98.4	305	99.7	
Overall morbidity	Yes	869	23.8	162	19.5	255	23.9	301	27.8	79	21.5	72	23.5	0.001
	No	2,784	76.2	668	80.5	813	76.1	781	72.2	288	78.5	234	76.5	
^b^ Major morbidity	Yes	223	6.1	22	3.0	69	6.5	87	8.0	25	6.8	17	5.6	0.000
	No	3,430	93.9	805	97.0	999	93.5	995	92.0	342	93.2	289	94.4	
Mortality	Yes	23	0.6	4	0.5	5	0.5	8	0.7	4	1.1	2	0.6	0.701
	No	3,630	99.4	826	99.5	1,063	99.5	1,074	99.3	363	98.9	304	99.4	

Abbreviations: ASA, American Society of Anesthesiologists.; BT, blood transfusion(s); CS, circular stapler; ERP, Enhanced recovery pathway.

^a^ Chi square independence test with four degrees of freedom.

^b^ Major: any event grade > II according to Clavien‐Dindo. Platforms were defined according to staple‐line architecture and mechanical design; detailed manufacturer and model information is provided in Table 2.

### Circular stapler platforms

Detailed information on circular stapler platforms, manufacturers and models used for end‐to‐end colorectal anastomosis is provided in Table 2. Platforms were classified according to staple‐line architecture and mechanical design. Manufacturer and model information is reported solely for descriptive and reproducibility purposes.

**TABLE 2:** Circular stapler platforms, manufacturers and models used for end‐to‐end colorectal anastomosis.

Circular stapler platform	Staple‐line architecture	Manufacturer	Model(s)	No. of patients (%)
Platform 1–3‐row circular stapler (n. = 830)	Three circular rows of B‐shaped staples varying in height	Covidien	TRIEEA28MT	190 (5.2)
			TRIEEA28XT	81 (2.2)
			TRIEEA31MT	412 (11.3)
			TRIEEA31XT	95 (2.6)
			TRIEEA33MT	45 (1.2)
			TRIEEA33XT	7 (0.2)
Platform 2–2‐row circular stapler (n. = 1,068)	Two circular rows of B‐shaped staples	Covidien	DSTEEA28	561 (15.3)
			DSTEEA28XL	78 (2.1)
			DSTEEA31	321 (8.8)
			DSTEEA31XL	37 (1.0)
			DSTEEA33	17 (0.5)
			DSTEEA33XL	54 (1.5)
Platform 3–2‐row circular stapler (n. = 1,082)	Two circular rows of B‐shaped staples	Ethicon	CDH29A	841 (23.0)
			CDH33A	72 (2.0)
			ECS29A	165 (4.5)
			ECS33A	4 (0.1)
Platform 4–2‐row powered circular stapler (n. = 367)	Two circular rows of staples with three‐dimensional architecture	Ethicon	CDH29P	292 (8.0)
			CDH31P	75 (2.1)
Platform 5–2‐row circular stapler (n. = 306)	Two circular rows of B‐shaped staples	Touchstone	CSC29A	136 (3.7)
			CSC33A	24 (0.7)
			ECSC29	131 (3.6)
			ECSC33	15 (0.4)

*Note*: Platforms were defined according to staple‐line architecture and mechanical design. Manufacturer and model information is provided for descriptive and reproducibility purposes only.

### Data management and ethics

Details of data collection procedures and ethical approval are provided in the Supporting Information. All data were prospectively collected, and the study was conducted in accordance with the Declaration of Helsinki and Good Clinical Practice guidelines, with approval from the relevant ethics committees.

### 60‐day adverse events

All enrolled patients were followed for 60 days after surgery. Adverse events were recorded and graded according to the Clavien‐Dindo classification [21], the Japanese Clinical Oncology Group (JCOG) extended criteria [22] and included any unplanned readmission, reoperation or death within 60 days after surgery. Anastomotic leakage (AL) was defined according to the international consensus [23], and anastomotic bleeding (AB) was defined as persistent rectal bleeding associated with a ≥ 20 g/L decrease in haemoglobin concentration [24].

### Outcomes

All outcomes were assessed 60 days after surgery. The primary endpoint was Overall AL (any AL). Secondary endpoints were Major AL (any AL grade > II), Overall AB (any AB), Major AB (any AB grade > II), Global morbidity (any adverse event), Major morbidity (any adverse event grade > II) and mortality (all‐cause death).

### Statistical analysis

No missing data were present in the final analytic dataset of 3,653 patients. The estimand of interest was the average treatment effect on the treated (ATT), representing the expected difference in outcomes among patients receiving the reference circular stapler platform had they instead received an alternative platform. The reference platform was selected based on findings from a previously published study [8].

Confounding was addressed using propensity score weighting in pairwise comparisons between the reference platform and each alternative platform. Propensity scores were estimated using GBM, a machine learning approach that flexibly captures complex, nonlinear relationships between treatment assignment and covariates while minimizing model misspecification [25]. Analyses were performed using the *twang* package in R (version 4.5.0).

Twenty‐one covariates reflecting patient‐, procedure‐ and centre‐level characteristics were selected a priori and corresponded to the descriptive variables (Figure 2). Effective sample sizes after weighting were evaluated to assess the stability of the weighted comparisons.

Treatment effects were estimated using doubly robust weighted outcome models adjusted for the same covariates included in the propensity score models. Weighted event rates represent marginal risk estimates in the weighted pseudo‐population. Sensitivity to unmeasured confounding was assessed using E‐values [26]. Additional methodological details are provided in the Supporting Information.

## RESULTS

### Association between circular stapler platform and postoperative outcomes

Among the 3,653 patients included in the final analytic cohort, overall anastomotic leakage occurred in 2.7% of patients receiving the reference 3‐row circular stapler platform. Before adjustment, higher crude rates of overall AL were observed for 2‐row platforms. After GBM weighting and doubly robust adjustment, Platforms 3 and 4 remained independently associated with a significantly increased risk of overall AL compared with the reference platform (Platform 3: weighted event rate 5.7%, OR 2.34, 95% CI 1.16–4.71; Platform 4: 6.6%, OR 2.81, 95% CI 1.16–6.80). No statistically significant differences were observed for Platforms 2 or 5 after weighting.

Among the measured covariates, residual imbalance after weighting was greatest for circular stapler diameter, particularly in comparisons involving Platforms 3 and 5, reflecting limited overlap in diameter distributions across commercially available stapler platforms (Table S4). This finding indicates constrained covariate overlap for certain platform–diameter combinations, consistent with the practical limitations of achieving full exchangeability in these comparisons.

Major anastomotic leakage was infrequent across all platforms. After weighting, no platform was associated with a statistically significant increase in major AL compared with the reference platform.

Overall, anastomotic bleeding was significantly more frequent with several 2‐row platforms. After GBM weighting, Platforms 2, 3 and 5 were associated with a markedly increased risk of overall anastomotic bleeding compared with the reference platform (ORs ranging from 5.65 to 7.23), with large E‐values indicating robustness to potential unmeasured confounding. Similar patterns were observed for major anastomotic bleeding, particularly for Platforms 3 and 4, which showed substantially higher adjusted odds despite the low absolute event rates. Given the small number of events, particularly for major bleeding, these estimates should be interpreted cautiously because of limited statistical stability and precision, and are better interpreted as indicating the direction rather than the exact magnitude of the association.

Overall morbidity did not differ significantly across stapler platforms after adjustment. In contrast, major morbidity remained significantly higher for Platforms 3 (weighted event rate 7.5%, OR 2.66, 95% CI 1.31–5.39) and 5 (8.4%, OR 2.95, 95% CI 1.30–6.72) compared with the reference platform. Thirty‐ and 60‐day mortality rates were low (<1%) and did not differ significantly across platforms.

Adjusted weighted event rates, odds ratios, 95% confidence intervals and E‐values for all primary and secondary endpoints are presented in Table 3.

**TABLE 3:** Adjusted logistic multiple regression analysis for primary and secondary endpoints before and after generalized boosted model (GBM) weighting.

Endpoints	Circular staplers	Before ^a^ GBM weighting (3,653 patients)			After ^a^ GBM weighting (3,653 patients)			
		Event rate (%)	^b^ OR (95% CI)	*p*	^c^ GBM‐weighted event rate (%)	^b^ OR (95% CI)	*p*	E‐value^d^
**Primary endpoint**								
Overall anastomotic leakage	^e^ Platform 1–3‐row	2.7	reference		2.7	reference		
	^f^ Platform 2–2‐row	4.7	2.05 (1.19–3.52)	0.009	3.9	1.53 (0.80–2.96)	0.202	
	^g^ Platform 3–2‐row	6.8	3.11 (1.76–5.51)	0.000	5.7	2.34 (1.16–4.71)	**0.018**	4.10 (1.59–8.89)
	^h^ Platform 4–2‐row powered	5.2	2.20 (1.13–4.30)	0.020	6.6	2.81 (1.16–6.80)	**0.022**	5.06 (1.59–13.08)
	^i^ Platform 5–2‐row	3.9	1.90 (0.88–4.12)	0.102	3.2	1.50 (0.57–3.92)	0.412	
**Secondary endpoints**								
Major anastomotic leakage	^e^ Platform 1–3‐row	2.4	reference		2.4	reference		
	^f^ Platform 2–2‐row	3.8	1.80 (1.01–3.23)	0.048	3.7	1.68 (0.78–3.59)	0.184	
	^g^ Platform 3–2‐row	5.5	2.59 (1.40–4.80)	0.003	4.5	2.03 (0.94–4.34)	0.068	
	^h^ Platform 4–2‐row powered	4.1	1.98 (0.96–4.12)	0.066	3.6	1.74 (0.61–5.01)	0.304	
	^i^ Platform 5–2‐row	2.6	1.31 (0.54–3.19)	0.550	1.8	0.85 (0.26–2.84)	0.795	
Overall anastomotic bleeding	^e^ Platform 1–3‐row	1.6	reference		1.6	reference		
	^f^ Platform 2–2‐row	4.1	3.50 (1.73–7.09)	0.000	5.5	6.83 (2.54–18.37)	**0.000**	13.13 (4.51–36.23)
	^g^ Platform 3–2‐row	4.0	3.64 (1.71–7.78)	0.001	3.8	5.65 (1.79–17.85)	**0.003**	10.78 (2.97–35.19)
	^h^ Platform 4–2‐row powered	2.2	1.92 (0.74–4.98)	0.182	2.6	2.68 (0.59–12.15)	0.202	
	^i^ Platform 5–2‐row	6.2	4.86 (2.13–11.09)	0.000	6.0	7.23 (2.56–20.47)	**0.000**	13.95 (4.55–40.42)
Major anastomotic bleeding	^e^ Platform 1–3‐row	0.4	reference		0.4	reference		
	^f^ Platform 2–2‐row	2.1	11.18 (2.68–46.60)	0.001	1.9	9.85 (1.63–59.67)	**0.013**	19.18 (2.63–118.84)
	^g^ Platform 3–2‐row	1.6	8.20 (1.83–36.79)	0.006	3.0	22.76 (2.94–176.16)	**0.003**	45.02 (5.33–351.83)
	^h^ Platform 4–2‐row powered	1.6	8.90 (1.79–44.22)	0.008	2.3	12.99 (1.26–133.41)	**0.031**	25.47 (1.84–266.32)
	^i^ Platform 5–2‐row	0.3	1.15 (0.10–13.23)	0.912	0.7	3.74 (0.25–56.88)	0.342	
Global morbidity	^e^ Platform 1–3‐row	19.5	reference		19.5	reference		
	^f^ Platform 2–2‐row	23.9	1.30 (1.02–1.66)	0.032	21.8	1.18 (0.86–1.63)	0.304	
	^g^ Platform 3–2‐row	27.8	1.59 (1.23–2.07)	0.001	23.0	1.31 (0.86–2.00)	0.214	
	^h^ Platform 4–2‐row powered	21.5	1.14 (0.82–1.57)	0.443	21.9	1.21 (0.72–2.01)	0.472	
	^i^ Platform 5–2‐row	23.5	1.25 (0.89–1.76)	0.203	23.7	1.23 (0.76–2.00)	0.393	
Major morbidity	^e^ Platform 1–3‐row	3.0	reference		3.0	reference		
	^f^ Platform 2–2‐row	6.5	2.32 (1.41–3.83)	0.001	4.5	1.50 (0.83–2.71)	0.182	
	^g^ Platform 3–2‐row	8.0	2.78 (1.65–4.70)	0.000	7.5	2.66 (1.31–5.39)	**0.007**	4.76 (1.96–10.25)
	^h^ Platform 4–2‐row powered	6.8	2.27 (1.24–4.16)	0.008	6.0	2.18 (0.92–5.19)	0.078	
	^i^ Platform 5–2‐row	5.6	1.94 (0.99–3.82)	0.054	8.4	2.95 (1.30–6.72)	**0.010**	5.35 (1.92–12.92)
Mortality	^e^ Platform 1–3‐row	0.5	reference		0.5	reference		
	^f^ Platform 2–2‐row	0.5	0.78 (0.18–3.31)	0.736	0.3	0.59 (0.11–3.28)	0.548	
	^g^ Platform 3–2‐row	0.7	1.02 (0.24–4.23)	0.981	0.4	0.49 (0.10–2.36)	0.370	
	^h^ Platform 4–2‐row powered	1.1	1.87 (0.40–8.77)	0.428	0.8	1.73 (0.32–9.28)	0.525	
	^i^ Platform 5–2‐row	0.7	1.23 (0.19–8.00)	0.831	0.8	1.17 (0.15–8.91)	0.883	

*Note:* The bold values indicate the statistically significant *p* values.

Abbreviation: CS: circular stapler.

^a^ GBM weighting: Generalized Boosted Model weighting.

^b^ OR (95%CI): odds ratio and 95% confidence intervals.

^c^ GBM‐weighted event rate (%): GBM‐weighted event rates represent adjusted marginal risk estimates in the ATT‐weighted pseudo‐population.

^d^ E‐values are only reported for statistically significant associations. Their absence indicates that the corresponding comparison was not statistically significant after GBM weighting and doubly robust adjustment.

^e^ Platform 1–3‐row CS: Covidien EEA™ circular stapler with Tri‐Staple™ Technology TRIEEA28MT, TRIEEA28XT, TRIEEA31MT, TRIEEA31XT, TRIEEA33MT, TRIEEA33XT.

^f^ Platform 2–2‐row CS: Covidien EEA™ circular stapler with DST™ Technology DSTEEA28, DSTEEA28XL, DSTEEA31, DSTEEA31XL, DSTEEA33, DSTEEA33XL.

^g^ Platform 3–2‐row CS: Ethicon circular stapler CDH29A, CDH33A, ECS29A, ECS33A.

^h^ Platform 4–2‐row powered CS: Ethicon Echelon Circular™ Powered Stapler CDH29P, CDH31P.

^i^ Platform 5–2‐row CS: Touchstone circular stapler CSC29A, CSC33A, ECSC29, ECSC33. Platforms were defined according to staple‐line architecture and mechanical design. Manufacturer and model information is reported for descriptive and reproducibility purposes only.

## DISCUSSION

The present multicentre analysis provides a comparative evaluation of circular stapler platforms in left colectomy and high anterior resection using an advanced multi‐treatment propensity‐based framework. After extensive adjustment for patient‐, procedure‐ and centre‐level characteristics, clinically relevant differences in anastomotic outcomes were observed across stapler platforms. Compared with the reference platform, Platforms 3 and 4 were associated with a significantly increased risk of overall anastomotic leakage, whereas several platforms were associated with an increased risk of anastomotic bleeding. These associations persisted after GBM weighting, were supported by high E‐values and were accompanied by higher rates of major postoperative morbidity, whereas no differences in short‐term mortality were observed.

This analysis addresses a device‐specific comparative effectiveness question that was not a primary or secondary endpoint of the iCral studies. Although part of the dataset derives from the ongoing iCral‐4 study, the present analysis was conceived independently to address a distinct clinical question using advanced causal inference methods applied to pooled individual patient‐level data.

### Comparison with existing literature

The impact of circular stapler technology on anastomotic outcomes has been extensively investigated in observational studies and meta‐analyses, but the evidence remains inconsistent. Most comparative studies evaluating two‐row versus three‐row circular staplers have reported either lower anastomotic leakage rates or a numerical trend favouring three‐row devices [4, 5, 8, 10], although these findings have varied according to study design and methods of confounding adjustment. In contrast, large administrative database analyses have reported neutral results, further highlighting the heterogeneity of the available evidence [7].

Recent meta‐analyses have attempted to synthesize this literature but have produced partly divergent conclusions depending on how stapler technology was defined and grouped. While some analyses suggest a reduction in anastomotic leakage associated with three‐row or powered devices [15, 16, 18], others [17] have reported no significant differences between three‐row and two‐row staplers. These divergent conclusions largely reflect differences in the studies included, which varied substantially in design, patient populations and statistical methodology.

Importantly, most available evidence evaluates individual device characteristics (e.g. staple‐row configuration or powered firing) or selected pairwise comparisons rather than complete commercial stapler platforms as used in routine clinical practice. Consequently, the relative contribution of individual engineering features cannot be disentangled from the combined effects of device design, surgical technique and perioperative care.

Within this context, the present study addresses this gap by comparing multiple commercially available stapler platforms within a unified analytical framework, thereby reflecting real‐world clinical practice rather than isolated technical attributes.

### Stapler platform and anastomotic leakage

In the present study, Platforms 3 and 4 remained associated with more than a twofold increase in overall anastomotic leakage after GBM weighting and doubly robust adjustment. Effect estimates were attenuated after weighting, indicating that part of the crude association was explained by baseline imbalances. Nevertheless, the persistence of the association after extensive adjustment suggests robustness to measured confounding, although residual unmeasured confounding, limited covariate overlap (particularly for stapler diameter) and variation in postoperative surveillance across centres may still have influenced the results.

The increased risk was confined to overall rather than major anastomotic leakage, a finding that warrants cautious interpretation because it may reflect differential ascertainment of Grade I leaks, differences in statistical power, leak severity or postoperative detection rather than a true dissociation between overall and major AL.

E‐value analyses indicated that modest unmeasured confounding could attenuate the observed associations, whereas substantially stronger confounding would be required to fully explain them.

Potential sources of residual confounding include surgeon‐specific technical expertise and intraoperative decision‐making, such as vascular ligation level, splenic flexure mobilization, rectal stump management, intraoperative assessment of anastomotic integrity and reinforcement strategies, particularly if unevenly distributed across stapler platforms. Because these factors are closely interrelated, they may function as confounders, mediators or proxies of surgical quality depending on their temporal relationship to stapler platform selection and anastomotic construction. Although individual intraoperative variables are recognized determinants of anastomotic integrity, their contribution to residual confounding cannot be directly inferred because these practices were not uniformly adopted across participating centres. Moreover, in centres using multiple stapler platforms, these interdependent intraoperative factors may influence stapler platform selection differently according to local practice and surgeon preference, precluding assumptions about the direction of any resulting residual confounding.

From a statistical standpoint, covariate balance improved substantially after GBM weighting, although limited covariate overlap persisted for Platform 3, resulting in the smallest effective sample size. Consequently, although the association with overall anastomotic leakage remained statistically significant, the corresponding effect estimate should be interpreted cautiously because of its reduced precision and confirmed in independent datasets.

### Anastomotic bleeding

After GBM weighting, several 2‐row platforms remained associated with an increased risk of overall and major anastomotic bleeding. However, these estimates were based on few events, particularly for major bleeding, resulting in wide confidence intervals. Accordingly, they should be interpreted cautiously as indicating the direction rather than the precise magnitude of the observed associations.

Experimental evidence suggests that graduated‐height staple configurations may improve staple‐line microvascular perfusion [27], providing a plausible biological explanation for the observed associations. However, the present study was not designed to identify which engineering characteristics, if any, account for these associations. Consequently, the findings should be regarded as hypothesis‐generating rather than as evidence of a specific causal biological mechanism.

### Impact on major morbidity

Although overall postoperative morbidity did not differ significantly across stapler platforms after adjustment, major morbidity remained significantly higher for Platforms 3 and 5, supporting the clinical relevance of the observed differences in anastomotic complications.

### Strengths & limitations

This study has several limitations. First, the analysis is based on pooled individual patient‐level data from multiple iCral studies conducted over different time periods, with approximately three quarters of the cohort derived from the published iCral‐2 and iCral‐3 studies and the remainder from the iCral‐4 study, whose primary results are currently under peer review. This approach was adopted to provide adequate statistical power for a comparative effectiveness analysis of relatively infrequent but clinically important outcomes, such as anastomotic leakage and bleeding. Comparative characteristics of the constituent cohorts and a sensitivity analysis restricted to the published iCral‐2 and iCral‐3 datasets are provided in the Supporting Information, showing consistent findings despite the smaller sample size.

Second, this post hoc comparative observational analysis is based on non‐random allocation of stapler platforms and a highly selected study population. Exclusion of urgent surgery, ASA class IV–V patients, protective stomas, ultra‐low rectal anastomoses and neoadjuvant therapy was intended to improve internal validity by reducing major sources of clinical heterogeneity and confounding. Consequently, the findings may not be generalizable to higher‐risk colorectal surgery populations.

Third, inclusion of both benign and malignant cases represents a potential source of confounding. However, surgery for malignancy was included as a prespecified covariate in the GBM, allowing comparisons between stapler platforms to be adjusted for the underlying disease indication.

Fourth, despite the use of advanced causal inference techniques, residual confounding due to unmeasured variables, including surgeon‐specific technical expertise and intraoperative decision‐making, cannot be completely excluded. Relevant intraoperative practices, such as vascular ligation level, splenic flexure mobilization, rectal stump management, perfusion assessment and air leak testing, were not uniformly adopted across participating centres and therefore could not be consistently incorporated into the adjusted analyses. In addition, although weighting substantially improved balance across most measured covariates, residual imbalance persisted for selected variables, particularly stapler diameter, reflecting limited covariate overlap between commercially available stapler platforms and their predominant diameter configurations. Consequently, the estimated effects should be interpreted as platform‐level associations observed in real‐world clinical practice rather than as diameter‐independent mechanical effects.

Fifth, the effective sample size after weighting was smallest for Platform 3, reflecting limited covariate overlap with the reference platform. Although the association with overall anastomotic leakage remained statistically significant, the corresponding effect estimate should be interpreted cautiously because of its reduced precision and confirmed in independent datasets.

## CONCLUSIONS

In this large multicentre observational analysis, selected circular stapler platforms were associated with clinically meaningful differences in the risk of anastomotic leakage. Commercial stapler platforms should not be considered fully interchangeable in real‐world clinical practice. The observed associations are more appropriately interpreted as platform‐level associations that may reflect the combined influence of multiple engineering characteristics and procedural factors rather than a single design feature. These findings apply to the selected study population and should not be extrapolated to patients at the highest baseline risk of anastomotic leakage. Although these results do not justify recommending one commercial platform over another in all clinical settings, they support careful consideration of stapler platform selection.

## AUTHOR CONTRIBUTIONS

**Francesco Masedu:** Conceptualization; methodology; software; data curation; investigation; validation; formal analysis; supervision; writing – original draft; writing – review and editing. **Stefano Guadagni:** Conceptualization; methodology; data curation; investigation; validation; supervision; visualization; writing – original draft; writing – review and editing. **Marco Catarci:** Conceptualization; methodology; investigation; validation; data curation; supervision; visualization; project administration; resources; writing – original draft; writing – review and editing.

## FUNDING INFORMATION

This research received no specific grants from any funding agency in the public, commercial or not‐for‐profit sectors.

## CONFLICT OF INTEREST STATEMENT

The authors declare no conflicts of interest.

## ETHICS STATEMENT

All data were prospectively collected, and the study was conducted in accordance with the Declaration of Helsinki and Good Clinical Practice guidelines, with approval from the relevant ethics committees (Marche Regional Ethics Committee [CERM] 2018/334 for iCral 2; CERM 2020/192 for iCral 3; Lazio 2 Ethics Committee 0054579/2022 for iCral 4).

## CONSENT

All patients enrolled in the prospective studies gave full written consent.

## Supporting information


**Table S1.** Comparison of the constituent iCral cohorts. To improve transparency regarding the contribution of the previously unpublished iCral‐4 dataset, the principal characteristics of the published iCral‐2/iCral‐3 cohorts are compared with those of the iCral‐4 cohort, including study period, sample size, overall anastomotic leakage rate, circular stapler platform distribution, participating centres and Enhanced Recovery Pathway (ERP) adherence.
**Table S2**. Sensitivity analysis restricted to the published iCral‐2 and iCral‐3 cohorts (*n* = 2,751). Treatment effect estimates for overall anastomotic leakage obtained after exclusion of the previously unpublished iCral‐4 cohort, compared with the primary pooled analysis.
**Table S3**. Definitions of the 20 Enhanced Recovery Pathway (ERP) items adopted in the present study.
**Table S4**. Post‐weighting Kolmogorov–Smirnov (KS) statistics and standardized mean differences (SMDs) for all covariates in each pairwise comparison between the reference Platform 1 and the alternative circular stapler platforms.

## Data Availability

The data that support the findings of this study are available on request from the corresponding author. The data are not publicly available due to privacy or ethical restrictions.
