# Supplementary material for: Circular stapler platform and the risk of anastomotic leakage after elective left colectomy and high anterior resection
Source: Colorectal Dis. 2026 Aug 3;28(8):e70564. doi: 10.1111/codi.70564 (PMC13434361; doi:10.1111/codi.70564)
Supplement: Supplementary file 1 [file CODI-28-0-s001.docx]

**Title: Circular Stapler Platform and the Risk of Anastomotic Leakage After Elective Left Colectomy and High Anterior Resection**

Authors: Stefano Guadagni^1,α^, MD, Marco Catarci^2,α,β^, MD, FACS, Francesco Masedu^3^, PhD; the Italian ColoRectal Anastomotic Leakage (iCral) study group*.

From the ^1^General Surgery Unit and Department of Biotechnological and Applied Clinical Sciences, Università degli Studi dell’Aquila, L’Aquila, Italy; e-mail: stefano.guadagni@univaq.it.

^2^General Surgery Unit, Sandro Pertini Hospital, ASL Roma 2, Roma, Italy; e-mail: marcocatarci@gmail.com

^3^Department of Biotechnological and Applied Clinical Sciences, Università degli Studi dell’Aquila, L’Aquila, Italy; e-mail: francesco.masedu@univaq.it

^α^Stefano Guadagni and Marco Catarci share the first authorship.

^β^Study group coordinator.

**Corresponding author.** Francesco Masedu. **Address**: Dipartimento di Scienze Cliniche Applicate e Biotecnologiche; Università degli Studi dell’Aquila, L’Aquila, Italy; Via Vetoio, snc; 67100 L’Aquila, Italy. **ORCID ID**: 0000-0003-0290-5324

* iCral study group investigators are: Marco Clementi^1^, MD, Paolo Ciano^2^, MD, Michele Benedetti^2^, MD, Leonardo Antonio Montemurro^2^, MD, Fanny Massimi^2^, MD, Elena Belloni^2^, MD, Matteo Di Carlo^2^, MD, Giacomo Ruffo^3^, MD, Elisa Bertocchi^3^, MD, Gaia Masini^3^, MD, Massimo Giuseppe Viola^4^, MD, Amedeo Altamura^4^, MD, Francesco Rubichi^4^, MD, Gianluca Garulli^5^, MD, Daniele Parlanti^5^, MD, Gabriele Vago^5^, MD, Carlo Di Marco^6^, MD, Marco Scatizzi^7^, MD, Lorenzo Pandolfini^7^, MD, Alessandro Falsetto^7^, MD, Vincenzo Bottino^8^, MD, Antonio Ferronetti^8^, MD, Ferdinando Ficari^9^, MD, Francesco Giudici^9^, MD, Fabio Cianchi^9^, MD, Paolo Delrio^10^, MD, Massimiliano Di Marzo^10^, MD, Felice Pirozzi^11^, MD, Antonio Sciuto^11^, MD, Maria Carmela Giuffrida^12^, MD, Marco Migliore^12^, MD, Raffaele De Luca^13^, MD, Michele Simone^13^, MD, Alberto Patriti^14^, MD, Valerio Sisti^14^, MD, Marcella Lodovica Ricci^14^, MD, Walter Siquini^15^, MD, Alessandro Cardinali^15^, MD, Stefano D’Ugo^16^, MD, Marcello Spampinato^16^, MD, Stefano Scabini^17^, MD, Alessandra Aprile^17^, MD, Domenico Soriero^17^, MD, Marco Caricato^18^, MD, FACS, Gabriella Teresa Capolupo^18^, MD, FACS, Giusto Pignata^19^, MD, Jacopo Andreuccetti^19^, MD, Ilaria Canfora^19^, MD, Andrea Liverani^20^, MD, Giuseppe Lamacchia^20^, MD, Claudia Franceschilli^20^, MD, Roberto Campagnacci^21^, MD, Angela Maurizi^21^, MD, Pierluigi Marini^22^, MD, Grazia Maria Attinà^22^, MD, Ugo Elmore^23^, MD, Lorenzo Gozzini^23^, MD, Francesco Corcione^24^, MD, Umberto Bracale^24^, MD, Roberto Peltrini^24^, MD, Maria Michela Di Nuzzo^24^, MD, Roberto Santoro^25^, MD, Pietro Amodio^25^, MD, Massimo Carlini^26^, MD, FACS, Domenico Spoletini^26^, MD, Rosa Marcellinaro^26^, MD, Antonio Giuliani^27^, MD, Giovanni Del Vecchio^27^, MD, Mario Sorrentino^28^, MD, Massimo Stefanoni^28^, MD, Giovanni Ferrari^29^, MD, Carmelo Magistro^29^, MD, Diletta Cassini^30^, MD, Alberto Di Leo^31^, MD, Lorenzo Crepaz^31^, MD, Augusto Verzelli^32^, MD, Andrea Budassi^32^, MD, Giuseppe Sica^33^, MD, Giulia Bagaglini^33^, MD, Stefano Rausei^34^, MD, Silvia Tenconi^34^, MD, Davide Cavaliere^35^, MD, Leonardo Solaini^35^, MD, Giorgio Ercolani^35^, MD, Sarah Molfino^36^, MD, Marco Milone^37^, MD, Giovanni Domenico De Palma^37^, MD, Giovanni Ciaccio^38^, MD, Paolo Locurto^38^, MD, Giovanni Tebala^39^, MD, Antonio Di Cintio^39^, MD, Luigi Boni^40^, MD, FACS, Elisa Cassinotti^40^, MD, Stefano Mancini^41^, MD, Andrea Sagnotta^41^, MD, PhD, Mario Guerrieri^42^, MD, Monica Ortenzi^42^, MD, Roberto Persiani^43^, MD, Alberto Biondi^43^, MD, Andrea Lucchi^44^, MD, FACS, Giulia Vitali^44^, MD, Dario Parini^45^, MD, Maurizio De Luca^45^, MD, Antonino Spinelli^46^, MD, Francesco Carrano^46^, MD, Michele Genna^47^, MD, Francesca Fior^47^, MD, Andrea Coratti^48^, MD, Giuseppe Giuliani^48^, MD, Dario Scala^49^, MD, Graziella Marino^49^, MD, Andrea Muratore^50^, MD, Patrizia Marsanic^50^, MD, Umberto Rivolta^51^, MD, Giuliano Sarro^51^, MD, Micaela Piccoli^52^, MD, Francesca Pecchini^52^, MD, Carlo Talarico^53^, MD, Vincenzo Greco^53^, MD, Michele Motter^54^, MD, Giuseppe Tirone^54^, MD, Mauro Totis^55^, MD, Nicolò Tamini^55^, MD, Marco Braga^55^, MD, Franco Roviello^56^, MD, Roberto Piagnerelli^56^, MD, Alessandro Anastasi^57^, MD, Giuseppe Canonico^57^, MD, Gianluca Guercioni^58^, MD, Simone Cicconi^58^, MD, Irene Marziali^58^, MD, Giuseppe Maria Ettorre^59^, MD, Marco Colasanti^59^, MD, Mauro Montuori^60^, MD, Enrico Pinotti^60^, MD, Pierpaolo Mariani^61^, MD, Roberta Carminati^61^, MD, Nicolò de Manzini^62^, MD, Edoardo Osenda^62^, MD, Annibale Donini^63^, MD, Luigina Graziosi^63^, MD, Mariano Fortunato Armellino^64^, MD, Ciro De Martino^64^, MD, Lucio Taglietti^65^, MD, Silvia Ruggiero^65^, MD, Gabriele Anania^66^, MD, Matteo Chiozza^66^, MD, Mariantonietta Di Cosmo^67^, MD, Daniele Zigiotto^67^, MD, Carlo Vittorio Feo^68^, MD, Fioralba Pindozzi^68^, MD, Paolo Millo^69^, MD, Manuela Grivon^69^, MD, Corrado Pedrazzani^70^, MD, Cristian Conti^70^, MD, Silvio Guerriero^71^, MD, Lorenzo Organetti^71^, MD, Andrea Costanzi^72^, MD, Michela Monteleone^72^, MD, Nereo Vettoretto^73^, MD, Emanuele Botteri^73^, MD, Federico Marchesi^74^, MD, Giorgio Dal Monte^74^, MD, Massimo Basti^75^, MD, Diletta Frazzini^75^, MD, Graziano Longo^76^, MD, Simone Santoni^76^, MD, Moreno Cicetti^77^, MD, Gabriele La Gioia^77^, MD, Giuseppe Brisinda^78^, MD, Maria Michela Chiarello^78^, MD, Maria Cariati^78^, MD, Stefano Berti^79^, MD, Andrea Gennai^79^, MD, Ugo Pace^80^, MD, Silvia De Franciscis^80^, MD, Felice Borghi^81^, MD, Luca Pellegrino^81^, MD, Gianandrea Baldazzi^82^, MD, Gian Luca Baiocchi^36^, MD, FACS, Agata Borsella^83^, MD, Andrea Gattolin^84^, MD, Valentina Testa^84^, MD, Michele Masetti^85^, MD, Chiara Giordano^85^, MD, Leonardo Vincenti^86^, MD, Valeria Andriola^86^, MD, Massimiliano Fabozzi^87^, MD, Giovanna Ioia^87^, MD, Francesco Stanzione^88^, MD, Domenico Manzolillo^88^, MD, Gianluigi Moretto^89^, MD, FACS, Harmony Impellizzeri^89^, MD, Isacco Montroni^90^, MD, Giacomo Frascaroli^90^, MD, Massimiliano Di Paola^91^, MD, Alessandro Arturi^91^, MD, Luca Morelli^92^, MD, Gregorio Di Franco^92^, MD, Fausto Catena^93^, MD, Nicola Zanini^93^, MD, Salvatore Ramuscello^94^, MD, Silvia Mancini^94^, MD, Alessandro Carrara^95^, MD, Marinella Menegazzo^95^, MD.

From the ^1^General Surgery Unit, University Hospital, L’Aquila; ^2^General Surgery Unit, Sandro Pertini Hospital, ASL Roma 2; ^3^General Surgery Unit, IRCCS Sacro Cuore Don Calabria Hospital, Negrar di Valpolicella (VR); ^4^General Surgery Unit, Cardinale G. Panico Hospital, Tricase (LE); ^5^General Surgery Unit, Infermi Hospital, Rimini; ^6^General Surgery Unit, AULSS2 Marca Trevigiana, Conegliano Veneto (TV); ^7^General Surgery Unit, Santa Maria Annunziata & Serristori Hospital, Florence; ^8^General & Oncologic Surgery Unit, Evangelico Betania Hospital, Napoli; ^9^General Surgery and IBD Unit, Careggi University Hospital, Firenze; ^10^Colorectal Surgical Oncology, Istituto Nazionale per lo Studio e la Cura dei Tumori, “Fondazione Giovanni Pascale IRCCS-Italia”, Napoli; ^11^General Surgery Unit, ASL Napoli 2 Nord, Pozzuoli (NA); ^12^General & Oncologic Surgery Unit, Department of Surgery, Santa Croce e Carle Hospital, Cuneo; ^13^Department of Surgical Oncology, IRCCS Istituto Tumori “Giovanni Paolo II”, Bari; ^14^Department of Surgery, Marche Nord Hospital, Pesaro e Fano (PU); ^15^General Surgery Unit, S. Lucia Hospital, Macerata; ^16^General Surgery Unit, “V. Fazzi” Hospital, Lecce; ^17^General & Oncologic Surgery Unit, IRCCS “San Martino” National Cancer Center, Genova; ^18^Colorectal Surgery Unit, Policlinico Campus BioMedico, Roma; ^19^2nd General Surgery Unit 2, Spedali Civili di Brescia; ^20^General Surgery Unit, Regina Apostolorum Hospital, Albano Laziale (RM); ^21^General Surgery Unit, “C. Urbani” Hospital, Jesi (AN); ^22^General & Emergency Surgery Unit, San Camillo-Forlanini Hospital, Roma; ^23^Gastroenterologic Surgery Unit, IRCCS S. Raffaele Hospital, Milano; ^24^General Oncologic and Mininvasive Surgery Unit, “Federico II” University, Napoli; ^25^General Oncologic Surgery Unit, Belcolle Hospital, Viterbo; ^26^General Surgery Unit, S. Eugenio Hospital, ASL Roma 2; ^27^General Surgery Unit, S. Carlo Hospital, Potenza; ^28^General Surgery Unit, Latisana-Palmanova Hospital, Friuli Centrale University (UD); ^29^General Oncologic and Mininvasive Surgery Unit, Great Metropolitan Niguarda Hospital, Milano; ^30^General Surgery Unit, ASST Nord Milano, Sesto San Giovanni (MI); ^31^General and Minimally Invasive Surgery Unit, San Camillo Hospital, Trento; ^32^General Surgery Unit, Profili Hospital, Fabriano (AN); ^33^Minimally Invasive Surgery Unit, Policlinico Tor Vergata University Hospital, Roma; ^34^General Surgery Unit, Gallarate Hospital (VA); ^35^General & Oncologic Surgery Unit, AUSL Romagna, Forlì (FC); ^36^General Surgery Unit 3, Department of Clinical and Experimental Sciences, University of Brescia; ^37^General & Endoscopic Surgery Unit, “Federico II” University, Napoli;^38^General Surgery Unit, S. Elia Hospital, Caltanissetta; ^39^General Surgery Unit, S. Maria Hospital, Terni; ^40^General Surgery Unit, Fondazione IRCCS Ca’ Granda, Policlinico Maggiore Hospital, Milano; ^41^General & Oncologic Surgery Unit, San Filippo Neri Hospital, ASL Roma 1; ^42^Surgical Clinic, Torrette Hospital, University of Ancona; ^43^General Surgery Unit, Fondazione Policlinico Universitario Agostino Gemelli IRCCS, Roma; ^44^General Surgery Unit, “Ceccarini” Hospital, Riccione (RN); ^45^General Surgery Unit, S. Maria della Misericordia Hospital, Rovigo; ^46^Colorectal Surgery Unit, Humanitas University, Rozzano (MI); ^47^General & Bariatric Surgery Unit, University Hospital, Verona; ^48^General Surgery Unit, Misericordia Hospital, Grosseto; ^49^Abdominal Oncologic Surgery Unit, Basilicata Oncologic Hospital, Rionero in Vulture (PZ); ^50^General Surgery Unit, “E. Agnelli” Hospital, Pinerolo (TO); ^51^General Surgery Unit, Fornaroli Hospital, ASST Milano Ovest, Magenta (MI); ^52^General Surgery Unit, Civil Hospital, Baggiovara (MO); ^53^General Surgery Unit, Villa dei Gerani Hospital, Vibo Valentia (VV); ^54^2nd General Surgery Unit, S. Chiara Hospital, Trento; ^55^Colorectal Surgery Unit, San Gerardo Hospital, ASST Monza; ^56^Surgical Clinic, University of Siena; ^57^General Surgery Unit, San Giovanni di Dio Hospital, Firenze; ^58^General Surgery Unit, “C. e G. Mazzoni” Hospital, Ascoli Piceno; ^59^General & Transplant Surgery Unit, San Camillo-Forlanini Hospital, Roma; ^60^General & Mininvasive Surgery Unit, S. Pietro Hospital, Ponte San Pietro (BG); ^61^General Surgery Unit, Pesenti Fenaroli Hospital, Alzano Lombardo (BG); ^62^Surgical Clinic, University of Trieste; ^63^General & Emergency Surgery Unit, University of Perugia; ^64^General & Emergency Surgery Unit, S. Giovanni di Dio e Ruggi d’Aragona Hospital, Salerno; ^65^General Surgery Unit, ASST Valcamonica, Esine (BS); ^66^General & Laparoscopic Surgery Unit, University Hospital, Ferrara; ^67^General & Upper GI Surgery Unit, University Hospital, Verona; ^68^General Surgery Unit, Delta Hospital, Lagosanto (FE); ^69^General Surgery Unit, “U. Parini” Regional Hospital, Aosta; ^70^General & HPB Surgery Unit, University Hospital, Verona; ^71^General Surgery Unit, “F. Murri” Hospital, Fermo; ^72^General Surgery Unit, S. Leopoldo Hospital, Merate (LC); ^73^General Surgery Unit, Spedali Civili of Brescia, Montichiari (BS); ^74^Surgical Clinic, University of Parma; ^75^General Surgery Unit, Spirito Santo Hospital, Pescara; ^76^General Surgery Unit, Policlinico Casilino, Roma; ^77^General Surgery Unit, S. Maria della Misericordia Hospital, Urbino (PU); ^78^General Surgery Unit, San Giovanni di Dio Hospital, Crotone; ^79^General Surgery Unit, ASL 5 Liguria POLL, La Spezia; ^80^UOSD Chirurgia Robotica Addominale, Istituto nazionale Tumori IRCSS Fondazione G. Pascale, Napoli; ^81^UOC Chirurgia Oncologica – Istituto Tumori di Candiolo (TO) FPO – IRCCS; ^82^UOC Chirurgia Generale - Nuovo Ospedale di Legnano - Via Papa Giovanni II; 20025 Legnano (MI) - ASST Ovest Milanese; ^83^General Surgery Unit, ASST Cremona; ^84^General Surgery Unit, Ospedale Regina Montis Regalis – Mondovì (CN); ^85^General Surgery Unit, Ospedale S. Maria della Scaletta - Ausl Imola (BO); ^86^General Surgery Unit, Policlinico di Bari; ^87^Chirurgia Generale ed Oncologica P.O " A. Tortona" Pagani (SA); ^88^General and Oncologic Surgery Unit, Pineta Grande Hospital-Clinica Padre Pio Mondragone (CE); ^89^General Surgery Unit, Ospedale “Dott. P.Pederzoli”- Peschiera del Garda (VR); ^90^General and Colorectal Surgery Unit, Ospedale di Faenza (RV); ^91^General Surgery Unit, Fatebenefratelli Cassia Hospital, Roma; ^92^General Surgery Unit, Azienda Ospedaliero-Universitaria Pisana, Pisa; ^93^General Surgery Unit, “Maurizio Bufalini” Hospital, Cesena (FC); ^94^General Surgery Unit, Ospedale di Chioggia (VE); ^95^General Surgery Unit, Ospedale di Rovereto e Arco (TN).

**Supplementary Materials - Index**

| **Supplementary Methods** |  |
| --- | --- |
| Study rationale and transparency of the pooled dataset | *pag. 6* |
| Eligibility Criteria | *pag. 7* |
| Comparison of the constituent iCral cohorts | *pag. 8* |
| Sensitivity analysis restricted to the published iCral-2 and iCral-3 cohorts | *pag. 9* |
| Data Collection, Quality Control, and Ethics | *pag. 10* |
| Statistical Methods | *pag. 11* |
| **Supplementary Tables** |  |
| TableS1 | *pag. 17* |
| TableS2 | *pag. 18* |
| TableS3 | *pag. 19* |
| TableS4 | *pag. 20* |
| **References** | *pag. 22* |
|  |  |

**Supplementary Methods**

**Study rationale and transparency of the pooled dataset**

This study was a preplanned post hoc retrospective analysis of a prospectively collected, multicenter database. The present analysis pooled individual patient–level data from three consecutive studies of the Italian ColoRectal Anastomotic Leakage (iCral) study group (iCral-2, iCral-3, and iCral-4), all of which predefined anastomotic leakage as a study endpoint. The aim was to evaluate the association between circular stapler platform and the risk of anastomotic dehiscence.

The scientific hypothesis underlying the present study was conceived before initiation of patient enrolment in the iCral-4 study. Accordingly, the iCral Steering Committee decided that the manufacturer, model, and diameter of the circular stapler would be prospectively recorded for every enrolled patient, allowing subsequent classification of stapler platforms. The present study represents a preplanned, hypothesis-driven secondary analysis of these prospectively collected data and was not specified as a primary or secondary endpoint of the original iCral study protocols. As no modification to the study protocol, patient management, or data collection procedures was required, no amendment to the original ethical approvals or trial registration (NCT05227014) was necessary.

**Eligibility Criteria**

Inclusion and exclusion criteria for the iCral 2 (NCT03771456) and iCral 3 (NCT04397627) studies were as follows.

Inclusion criteria: 1.Adult patients undergoing laparoscopic, robotic, open, or converted ileo-colo-rectal resection with anastomosis, including planned Hartmann’s reversals; 2.American Society of Anesthesiologists (ASA) class I–III; 3.Elective or delayed-urgency surgery (defined as >48 hours from admission in iCral 2 and >24 hours from admission in iCral 3); 4. Written informed consent for study participation and processing of sensitive data.

Exclusion criteria: 1. Pregnancy; 2. Hyperthermic intraperitoneal chemotherapy (HIPEC) for carcinomatosis; 3. Incomplete data.

The iCral 2 study excluded patients with a protective stoma proximal to the anastomosis, whereas these patients were included in the iCral 3 study.

Inclusion and exclusion criteria for the iCral 4 study (NCT05227014) were as follows.

Inclusion criteria: 1. Adult patients undergoing laparoscopic, robotic, open, or converted ileo-colo-rectal resection with anastomosis, including planned Hartmann’s reversals; 2. ASA class I–IV; 3. Elective or delayed-urgency surgery (>24 hours from admission); 4. Written informed consent for study participation.

Exclusion criteria: 1. Pregnancy; 2. Hyperthermic intraperitoneal chemotherapy (HIPEC) for carcinomatosis; 3. ASA class V; 4. Emergent surgery (≤24 hours from admission).

**Comparison of the constituent iCral cohorts**

To improve transparency regarding the contribution of the unpublished iCral-4 dataset, **Table S1** compares the principal characteristics of the published iCral-2/iCral-3 cohorts with those of the iCral-4 cohort, including study period, sample size, overall anastomotic leakage rate, stapler platform distribution, center characteristics, and ERP adherence.

**Supplementary Table S1**

|  | **iCral-2 and iCral-3** | **iCral-4** | p* |
| --- | --- | --- | --- |
|  | (n.=2,751 - 2019/2021) | (n.=902 - 2022/2023) |  |
| **Overall anastomotic leakage rate** | 4.98 | 4.32 | .865 |
| **Platform distribution** |  |  |  |
| -1 (ref.) | 17.74 | 37.9 | .000 |
| -2 | 34.10 | 14.41 | .000 |
| -3 | 32.21 | 21.73 | .004 |
| -4 | 6.25 | 21.62 | .000 |
| -5 | 9.71 | 4.32 | .275 |
| **Center characteristics** |  |  |  |
| *Center volume* |  |  |  |
| < 4 cases/month | 23.81 | 27.49 | .253 |
| ≥ 4 cases/month) | 76.19 | 72.51 | .057 |
| *Center use of ≥ 2 circular stapler platforms* |  |  |  |
| < 2 | 7.42 | 6.76 | .862 |
| ≥ 2 | 92.58 | 93.24 | .522 |
| *Enhanced Recovery Pathway program items adherence* |  |  |  |
| ≥ 75 | 45.40 | 60.09 | .000 |

*Two sample T-test of proportions

The iCral-4 subgroup included 902 patients treated between 2022 and 2023 in 44 centers. Thirty-four of the 44 centers participating in iCral-4 had already participated in the published iCral-2/iCral-3 studies, indicating substantial continuity of the participating institutions. The overall anastomotic leakage rate was comparable between datasets (4.32% vs 4.98%, p=0.865), supporting the clinical consistency of the cohorts. As expected from the temporal evolution of clinical practice, the distribution of stapler platforms differed between study periods, with increased use of Platforms 1 and 4 and reduced use of Platforms 2 and 3 in iCral-4. ERP adherence also significantly increased in the more recent cohort. In contrast, center characteristics remained largely unchanged over time, with comparable distributions of hospital volume and use of multiple circular stapler platforms across the constituent cohorts. These variables were explicitly included among the prespecified covariates in the generalized boosted model propensity weighting, thereby accounting for temporal differences in the adjusted analyses.

**Sensitivity analysis restricted to the published iCral-2 and iCral-3 cohorts (n=2,751)**

The results of a sensitivity analysis restricted to the published iCral-2 and iCral-3 cohorts (n=2,751) are reported in Supplementary Table S2, in comparison to the primary analysis.

**Supplementary Table S2**

| **Endpoints** | **Circular staplers** | **After ^a^GBM weighting (2,751 patients)** | | | **After ^a^GBM weighting (3,653 patients)** | | |
| --- | --- | --- | --- | --- | --- | --- | --- |
|  |  | **^b^OR (95% CI)** | **p** | **E-Value*** | **^b^OR (95% CI)** | **p** | **E-Value*** |
| **Primary endpoint** | | | | | | | |
| Overall anastomotic leakage | ^c^Platform 1 - 3-row | reference |  |  | reference |  |  |
|  | ^d^Platform 2 - 2-row | 1.70 (0.77-3.77) | .189 |  | 1.53 (0.80-2.96) | .202 |  |
|  | ^e^Platform 3 - 2-row | 2.04 (0.89-4.67) | .092 |  | 2.34 (1.16-4.71) | **.018** | 4.10 (1.59-8.89) |
|  | ^f^Platform 4 - 2-row powered | 3.42 (1.09-10.68) | **.035** | 6.29 (1.41-20.86) | 2.81 (1.16-6.80) | **.022** | 5.06 (1.59-13.08) |
|  | ^g^Platform 5 - 2-row | 1.05 (0.35-3.12) | .935 |  | 1.50 (0.57-3.92) | .412 |  |

*^a^GBM weighting: Generalized Boosted Model weighting; ^b^OR (95%CI): odds ratio and 95% confidence intervals; CS: circular stapler; ^c^Platform 1 - 3-row CS:* *Covidien EEA^TM^ circular stapler with Tri-Staple ^TM^ Technology TRIEEA28MT, TRIEEA28XT, TRIEEA31MT, TRIEEA31XT, TRIEEA33MT, TRIEEA33XT; ^d^Platform 2 - 2-row CS: Covidien EEA^TM^ circular stapler with DST^TM^ Technology DSTEEA28, DSTEEA28XL, DSTEEA31, DSTEEA31XL, DSTEEA33, DSTEEA33XL; ^e^Platform 3 - 2-row CS: Ethicon circular stapler CDH29A, CDH33A, ECS29A, ECS33A; ^f^Platform 4 - 2-row powered CS: Ethicon Echelon Circular™ Powered Stapler CDH29P, CDH31P; ^g^Platform 5 - 2-row CS: Touchstone circular stapler CSC29A, CSC33A, ECSC29, ECSC33. Platforms were defined according to staple-line architecture and mechanical design. Manufacturer and model information is reported for descriptive and reproducibility purposes only. *E-values are only reported for statistically significant associations, and that absence of an E-value in a row implies the comparison was not statistically significant after weighting*

The sensitivity analysis demonstrated a high degree of consistency with the primary analysis. The direction and magnitude of the estimated treatment effects remained essentially unchanged. Platform 4 remained significantly associated with an increased risk of overall anastomotic leakage, whereas Platforms 2 and 5 remained non-significant. For Platform 3, the estimated effect size was very similar to that observed in the pooled analysis (OR 2.04 vs 2.34), although statistical significance was not reached because of the reduced sample size and corresponding loss of precision.

Overall, these findings indicate that inclusion of the iCral-4 cohort primarily improved statistical precision and power without materially changing the estimated treatment effects, thereby supporting the robustness of the main conclusions.

**Data Collection, Quality Control, and Ethics**

All data were prospectively entered into a secure, web-based database using a dedicated electronic case report form specifically designed for the three studies and accessible only through individual investigator credentials. Data collection included continuous and categorical variables related to patient demographics, comorbidities, surgical indication and technique, adherence to Enhanced Recovery Pathway items, circular stapler characteristics, and postoperative outcomes.

Data quality was ensured through systematic checks for consistency, plausibility, and completeness performed by local investigators and subsequently verified by the study coordinator. Any discrepancies were resolved through direct communication with the enrolling centers. During the perioperative period, patients were assessed daily by local investigators, who recorded and graded all adverse events. Decisions regarding complementary imaging and additional interventions were made according to local clinical practice.

All three studies were conducted in accordance with the Declaration of Helsinki and the Good Clinical Practice E6 (R2) guidelines. Study protocols were approved by the ethics committee of the coordinating center (Marche Regional Ethics Committee [CERM] 2018/334 for iCral 2; CERM 2020/192 for iCral 3; Lazio 2 Ethics Committee 0054579/2022 for iCral 4) and registered at ClinicalTrials.gov (NCT03771456, NCT04397627, and NCT05227014, respectively). Approval was subsequently obtained from the local ethics committees of all participating centers. All studies adhered to the Strengthening the Reporting of Cohort Studies in Surgery (STROCSS) guidelines. De-identified individual participant–level data are available upon reasonable request by contacting the study coordinator.

**Supplementary Statistical Methods**

**1. Sample Size Considerations**

No formal sample size calculation was performed for the present analysis, given the large number of included cases, which substantially exceeded commonly accepted requirements for multivariable logistic regression modeling^2^ and the rule of thumb of a minimum of 10 outcome events per explanatory variable.^3^ In addition, sample size calculations had already been performed and reported in the original iCral-2^4^and iCral-3^5^ studies.

**2. Estimand Definition**

The estimand of interest was the average treatment effect on the treated (ATT), corresponding to the causal question: “How would the average outcome change if patients receiving the reference treatment (end-to-end anastomosis using circular stapler platform 1) had instead received an alternative stapler platform (platforms 2, 3, 4, or 5)?”

The reference platform was selected based on findings from a previously published propensity score–matched analysis of the iCral-2 and iCral-3 cohorts.^6^

**3. Propensity Score Estimation**

Propensity score weighting was used to control for confounding in pairwise comparisons between the reference platform and each alternative platform. Propensity scores were estimated using generalized boosted models (GBM), which flexibly model complex and potentially nonlinear relationships between treatment assignment and covariates while limiting overfitting.^7^

GBM was selected because propensity scores estimated using GBM have consistently achieved superior covariate balance and lower bias than those estimated using conventional multinomial logistic regression in multiple-treatment settings.⁷ All analyses were performed using the “twang” package (Toolkit for Weighting and Analysis of Non-equivalent Groups) in R (version 4.5.0; R Foundation for Statistical Computing, Vienna, Austria).

**4. Model Specification and Balance Diagnostics**

The generalized boosted model (GBM) algorithm iteratively estimated propensity-score weights by minimizing differences in weighted covariate distributions across treatment groups relative to the reference Platform 1. Covariate balance was assessed over 20,000 iterations using the Kolmogorov–Smirnov mean (KS.mean) statistic, with a predefined threshold of 0.20 indicating negligible residual imbalance.

The KS.mean statistic was selected because it evaluates balance across the entire covariate distribution rather than only differences in covariate means, making it particularly suitable for generalized boosted model weighting. As propensity-score weights were estimated and optimized using the KS.mean stopping rule implemented in TWANG, KS statistics were considered the primary balance diagnostic. Standardized mean differences (SMDs) were additionally reported as complementary descriptive measures to facilitate interpretation and comparison with the broader propensity-score literature.

Post-weighting KS statistics and SMDs for all covariates in each pairwise comparison with the reference Platform 1 are provided in Supplementary Table S4.

**Supplementary Table S4**

| **Covariate** | **SMD*** | **KS**** | **Reference Platform 1** |
| --- | --- | --- | --- |
| Sex | -0.01 | 0.01 | Platform2 |
| Age | 0.02 | 0.01 | Platform2 |
| American Society of Anesthesiologists Class | 0.01 | 0.01 | Platform2 |
| Body Mass Index | 0.01 | 0 | Platform2 |
| Diabetes | 0.05 | 0.02 | Platform2 |
| Intra- and/or postoperative blood transfusion(s) | 0.01 | 0 | Platform2 |
| Preoperative blood transfusion(s) | 0.05 | 0.01 | Platform2 |
| Chronic renal failure | 0.1 | 0.02 | Platform2 |
| Chronic liver disease | 0.08 | 0.01 | Platform2 |
| Center volume | 0.04 | 0.01 | Platform2 |
| Operation length | 0.06 | 0.03 | Platform2 |
| Overall items ERP adherence | 0.05 | 0.02 | Platform2 |
| Preoperative items ERP adherence | -0.25 | 0.12 | Platform2 |
| Intraoperative items ERP adherence | -0.07 | 0.03 | Platform2 |
| Postoperative items ERP adherence | 0.06 | 0.03 | Platform2 |
| Surgery for malignancy | 0.01 | 0.01 | Platform2 |
| Open Surgery | 0 | 0 | Platform2 |
| Type of resection | 0.04 | 0.02 | Platform2 |
| Intracorporeal anastomosis | -0.02 | 0.01 | Platform2 |
| Diameter of circular stapler | 0.12 | 0.06 | Platform2 |
| Center use of ≥ 2 circular stapler platforms | 0.01 | 0 | Platform2 |
| Sex | -0.03 | 0.01 | Platform3 |
| Age | -0.07 | 0.04 | Platform3 |
| American Society of Anesthesiologists Class | 0 | 0 | Platform3 |
| Body Mass Index | 0.08 | 0.04 | Platform3 |
| Diabetes | 0.17 | 0.05 | Platform3 |
| Intra- and/or postoperative blood transfusion(s) | 0.08 | 0.01 | Platform3 |
| Preoperative blood transfusion(s) | 0.16 | 0.03 | Platform3 |
| Chronic renal failure | 0.09 | 0.02 | Platform3 |
| Chronic liver disease | 0.08 | 0.01 | Platform3 |
| Center volume | 0.03 | 0.01 | Platform3 |
| Operation length | 0.13 | 0.06 | Platform3 |
| Overall items ERP adherence | 0.01 | 0.01 | Platform3 |
| Preoperative items ERP adherence | -0.3 | 0.14 | Platform3 |
| Intraoperative items ERP adherence | 0.01 | 0.01 | Platform3 |
| Postoperative items ERP adherence | 0 | 0 | Platform3 |
| Surgery for malignancy | -0.21 | 0.11 | Platform3 |
| Open Surgery | -0.05 | 0.01 | Platform3 |
| Type of resection | 0.11 | 0.05 | Platform3 |
| Intracorporeal anastomosis | -0.1 | 0.03 | Platform3 |
| Diameter of circular stapler | 0.58 | 0.27 | Platform3 |
| Center use of ≥ 2 circular stapler platforms | -0.03 | 0 | Platform3 |
| Sex | 0.04 | 0.02 | Platform4 |
| Age | 0.03 | 0.02 | Platform4 |
| American Society of Anesthesiologists Class | 0.09 | 0.04 | Platform4 |
| Body Mass Index | -0.13 | 0.06 | Platform4 |
| Diabetes | 0.09 | 0.03 | Platform4 |
| Intra- and/or postoperative blood transfusion(s) | -0.01 | 0 | Platform4 |
| Preoperative blood transfusion(s) | 0.11 | 0.02 | Platform4 |
| Chronic renal failure | 0.06 | 0.01 | Platform4 |
| Chronic liver disease | 0.06 | 0.01 | Platform4 |
| Center volume | 0.14 | 0.05 | Platform4 |
| Operation length | -0.01 | 0 | Platform4 |
| Overall items ERP adherence | 0 | 0 | Platform4 |
| Preoperative items ERP adherence | -0.26 | 0.12 | Platform4 |
| Intraoperative items ERP adherence | -0.17 | 0.08 | Platform4 |
| Postoperative items ERP adherence | 0.07 | 0.03 | Platform4 |
| Surgery for malignancy | -0.08 | 0.04 | Platform4 |
| Open Surgery | 0.02 | 0 | Platform4 |
| Type of resection | 0.1 | 0.04 | Platform4 |
| Intracorporeal anastomosis | -0.01 | 0 | Platform4 |
| Diameter of circular stapler | 0.09 | 0.04 | Platform4 |
| Center use of ≥ 2 circular stapler platforms | -0.05 | 0 | Platform4 |
| Sex | 0 | 0 | Platform5 |
| Age | 0 | 0 | Platform5 |
| American Society of Anesthesiologists Class | 0.13 | 0.06 | Platform5 |
| Body Mass Index | 0.01 | 0 | Platform5 |
| Diabetes | 0.06 | 0.02 | Platform5 |
| Intra- and/or postoperative blood transfusion(s) | 0 | 0 | Platform5 |
| Preoperative blood transfusion(s) | 0.09 | 0.02 | Platform5 |
| Chronic renal failure | 0.01 | 0 | Platform5 |
| Chronic liver disease | 0.09 | 0.01 | Platform5 |
| Center volume | 0.19 | 0.07 | Platform5 |
| Operation length | -0.04 | 0.02 | Platform5 |
| Overall items ERP adherence | 0.51 | 0.22 | Platform5 |
| Preoperative items ERP adherence | 0.17 | 0.08 | Platform5 |
| Intraoperative items ERP adherence | 0.14 | 0.07 | Platform5 |
| Postoperative items ERP adherence | 0.44 | 0.2 | Platform5 |
| Surgery for malignancy | 0.16 | 0.08 | Platform5 |
| Open Surgery | 0.04 | 0 | Platform5 |
| Type of resection | 0.07 | 0.03 | Platform5 |
| Intracorporeal anastomosis | -0.15 | 0.04 | Platform5 |
| Diameter of circular stapler | 0.87 | 0.41 | Platform5 |
| Center use of ≥ 2 circular stapler platforms | 0.12 | 0.01 | Platform5 |

*SMD: Standardized mean difference; **KS: Kolmogorov–Smirnov mean.

Model parameters were specified as follows: a learning rate (shrinkage parameter) of 0.01, a maximum tree depth of 3, and up to 20,000 trees, beyond which no further improvement in covariate balance was observed. All remaining parameters followed the default settings of the twang package, which relies on the gbm library for gradient boosting and regularization.

**5. Covariate Selection and Rationale for ATT Weighting**

The 21 covariates included in the generalized boosted model (GBM) were selected a priori based on clinical relevance and their role in influencing treatment allocation among treated patients, consistent with estimation of the average treatment effect on the treated (ATT). Rather than restricting adjustment to baseline confounders alone, covariate selection was guided by a causal framework aimed at reducing residual confounding in a real-world observational setting, with effect estimates interpreted as total effects. Because both benign and malignant indications were included within the same analytical framework, surgery for malignancy was included as a balancing covariate in the GBM to ensure that comparisons between stapler platforms were adjusted for the underlying pathology.

*Baseline confounders.*

Age, sex, ASA class, body mass index, diabetes, chronic renal failure, and chronic liver failure were included as baseline confounders. These variables reflect baseline physiological reserve and comorbidity burden, precede treatment assignment, influence perioperative decision-making, and are independently associated with postoperative outcomes. Adjustment for these factors is essential to reduce confounding by indication and ensure comparability between treated patients and their weighted controls.

*Disease- and procedure-related confounders and proxies.*

Surgery for malignancy, left colectomy as resection type, and procedure duration were included to capture disease burden and operative complexity, which influence both treatment allocation and postoperative outcomes. Although procedure duration may be partially influenced by intraoperative management, it also serves as a proxy for operative difficulty and case complexity and was therefore retained in the model.

*Center-level structural confounders.*

Center volume and center use of ≥2 circular stapler platforms were incorporated as structural confounders to account for institutional practice patterns, procurement policies, implementation of enhanced recovery pathways (ERP), and differences in surgical expertise. These center-level factors strongly influence both treatment availability and preference and are therefore important for valid ATT estimation. The variable indicating use of ≥2 stapler platforms serves as a proxy for platform availability and institutional preference, although it does not fully account for surgeon-specific decision-making.

*Proxy mediators related to surgical technique.*

Open versus minimally invasive surgery, intra- versus extracorporeal anastomosis, and circular stapler diameter were included as proxy mediators. While these variables may lie partially on the causal pathway between stapler platform and outcome, they also reflect baseline patient risk, surgeon preference, and institutional norms that influence treatment allocation among treated patients. Their inclusion improves covariate balance and reduces residual confounding within the ATT framework.

*Blood transfusions as confounders and mediators.*

Preoperative blood transfusions were included as baseline confounders, as they occur prior to exposure assignment and reflect underlying anemia and disease severity that influence both treatment allocation and postoperative outcomes. In contrast, intraoperative and postoperative blood transfusions were considered potential post-exposure mediators, reflecting intraoperative complexity, bleeding, or early complications. Although these variables may lie on the causal pathway, their inclusion in the GBM was intended to improve covariate balance and reduce residual confounding in this observational setting. Given the ATT estimand, effect estimates are interpreted as total effects rather than direct effects.

*Enhanced Recovery Pathway variables.*

Preoperative, intraoperative, and postoperative ERP adherence were included as system-level indicators of perioperative care delivery and protocol implementation. Several individual ERP components (e.g., no bowel preparation, no nasogastric tube, no drain, postoperative bladder catheter removal) function as proxy mediators, while others (e.g., prehabilitation and minimally invasive surgery) are inherently collinear with other covariates. To mitigate multicollinearity and overparameterization, individual ERP items were not modelled separately; instead, composite ERP adherence scores were used. ERP variables were not intended to isolate causal mediation effects but to adjust for differences in the perioperative care context among treated patients.

Adherence to each individual ERP item was recorded as a binary variable (adherent vs non-adherent). For each patient, phase-specific and overall ERP adherence were calculated as the percentage of adhered items within each group. Median adherence percentages were then calculated across the study population. Based on these medians, four binary adherence variables were generated, classifying patients as having high adherence (≥ median) or low adherence (< median).

**6. Effective Sample Size**

The effective sample size (ESS) of each treatment group after weighting was estimated to assess the precision of the weighted comparisons. As expected under ATT weighting, the effective sample size of the reference platform remained essentially unchanged. The resulting effective sample sizes were 830 patients for the reference platform, 380.3 for Platform 2, 78.8 for Platform 3, 111.9 for Platform 4, and 135.2 for Platform 5.

**7. Outcome Modeling and Sensitivity Analysis**

All weighted outcome models were adjusted for the same prespecified 21 covariates included in the propensity score estimation, applying a doubly robust approach to estimate treatment effects.^7^ Weighted event rates represent marginal risk estimates in the ATT-weighted pseudo-population.

Sensitivity to potential unmeasured confounding was evaluated using the E-value, which quantifies the minimum strength of association that an unmeasured confounder would need to have with both treatment assignment and the outcome to fully explain the observed association.^8^

**8. Reproducibility**

All R code used for the statistical analyses is available upon reasonable request from the study coordinator.

**Supplementary Table S1**

|  | **iCral-2 and iCral-3** | **iCral-4** | p* |
| --- | --- | --- | --- |
|  | (n.=2,751 - 2019/2021) | (n.=902 - 2022/2023) |  |
| **Overall anastomotic leakage rate** | 4.98 | 4.32 | .865 |
| **Platform distribution** |  |  |  |
| -1 (ref.) | 17.74 | 37.9 | .000 |
| -2 | 34.10 | 14.41 | .000 |
| -3 | 32.21 | 21.73 | .004 |
| -4 | 6.25 | 21.62 | .000 |
| -5 | 9.71 | 4.32 | .275 |
| **Center characteristics** |  |  |  |
| *Center volume* |  |  |  |
| < 4 cases/month | 23.81 | 27.49 | .253 |
| ≥ 4 cases/month) | 76.19 | 72.51 | .057 |
| *Center use of ≥ 2 circular stapler platforms* |  |  |  |
| < 2 | 7.42 | 6.76 | .862 |
| ≥ 2 | 92.58 | 93.24 | .522 |
| *Enhanced Recovery Pathway program items adherence* |  |  |  |
| ≥ 75 | 45.40 | 60.09 | .000 |

*Two sample T-test of proportions

**Supplementary Table S2**

| **Endpoints** | **Circular staplers** | **After ^a^GBM weighting (2,751 patients)** | | | **After ^a^GBM weighting (3,653 patients)** | | |
| --- | --- | --- | --- | --- | --- | --- | --- |
|  |  | **^b^OR (95% CI)** | **p** | **E-Value*** | **^b^OR (95% CI)** | **p** | **E-Value*** |
| **Primary endpoint** | | | | | | | |
| Overall anastomotic leakage | ^c^Platform 1 - 3-row | reference |  |  | reference |  |  |
|  | ^d^Platform 2 - 2-row | 1.70 (0.77-3.77) | .189 |  | 1.53 (0.80-2.96) | .202 |  |
|  | ^e^Platform 3 - 2-row | 2.04 (0.89-4.67) | .092 |  | 2.34 (1.16-4.71) | **.018** | 4.10 (1.59-8.89) |
|  | ^f^Platform 4 - 2-row powered | 3.42 (1.09-10.68) | **.035** | 6.29 (1.41-20.86) | 2.81 (1.16-6.80) | **.022** | 5.06 (1.59-13.08) |
|  | ^g^Platform 5 - 2-row | 1.05 (0.35-3.12) | .935 |  | 1.50 (0.57-3.92) | .412 |  |

*^a^GBM weighting: Generalized Boosted Model weighting; ^b^OR (95%CI): odds ratio and 95% confidence intervals; CS: circular stapler; ^c^Platform 1 - 3-row CS: Covidien EEA^TM^ circular stapler with Tri-Staple ^TM^ Technology TRIEEA28MT, TRIEEA28XT, TRIEEA31MT, TRIEEA31XT, TRIEEA33MT, TRIEEA33XT; ^d^Platform 2 - 2-row CS: Covidien EEA^TM^ circular stapler with DST^TM^ Technology DSTEEA28, DSTEEA28XL, DSTEEA31, DSTEEA31XL, DSTEEA33, DSTEEA33XL; ^e^Platform 3 - 2-row CS: Ethicon circular stapler CDH29A, CDH33A, ECS29A, ECS33A; ^f^Platform 4 - 2-row powered CS: Ethicon Echelon Circular™ Powered Stapler CDH29P, CDH31P; ^g^Platform 5 - 2-row CS: Touchstone circular stapler CSC29A, CSC33A, ECSC29, ECSC33. Platforms were defined according to staple-line architecture and mechanical design. Manufacturer and model information is reported for descriptive and reproducibility purposes only. *E-values are only reported for statistically significant associations, and that absence of an E-value in a row implies the comparison was not statistically significant after weighting*

**Table S3** supplementary material: Definitions of the 20 Enhanced Recovery Pathway (ERP) items adopted in the present study.

| **ITEM** | | **Adherence criteria** |
| --- | --- | --- |
| Preoperative | Prehabilitation (Nutritional and Physical) | All patients were submitted to nutritional screening through Mini Nutritional Assessment Short Form (MNA-SF).^1^ All patients showing MNA-SF < 12 (malnourished or suspect malnutrition) and body mass index (BMI) > 30 (obesity) receive specific nutritional consultation. Patients receive a standard protocol of physical activity to be accomplished in the preoperative period; frail and limited motility patients are submitted to specific geriatrician/physiatrist consultation and personalized activity program |
|  | Counseling | Patient and his familiars/caregivers receive full information and suggestions regarding perioperative program from surgeon, anesthesiologist and case-manager |
|  | Preoperative Immunonutrition | Patient is administered Impact Oral ™ (Nestlè Health Science, Italy) 330 ml per os, three bricks per day during the 5 days preceding surgery or two bricks per day during the 7 days preceding surgery |
|  | Antithrombotic prophylaxis | Patient receives graduate compression stockings and/or pneumatic compression device, together with prophylaxis with low molecular weight heparin during the perioperative period, to be extended up to 28 days after surgery in case of malignancy |
|  | Antibiotic prophylaxis | Patients are administered i.v. antibiotic 30 to 60 minutes before incision, according to local protocols |
|  | No bowel preparation | No routine bowel preparation is used, except in case of anticipated need for covering stoma |
|  | Oral carbohydrates load | Carbohydrates rich beverage (12.5% maltrodextrins, PreOp™, Nutricia Italy) is given preoperatively (800 ml on the evening before surgery and 400 ml 2 to 3 hours before surgery) |
| Intraoperative | No premedication | No long- or medium-action sedatives. Short and ultra-short acting sedatives (e.g. Lorazepam, Midazolam, Methohexital, Dexmedetomidine, Ketamine) are allowed before performing spinal, epidural or loco-regional anesthesia |
|  | PONV prophylaxis | Postoperative nausea/vomiting (PONV) prophylaxis is administered according to individual risk assessment (Apfel score) through a multimodal approach |
|  | Normothermia | Body temperature is monitored during surgery, utilizing fluid warmers and/or thermic blankets as necessary |
|  | Standard anesthetic protocol | General anesthesia through short-acting anesthetics, cerebral activity monitoring to enhance recovery and to reduce postoperative delirium, anesthesia level monitoring and complete reversal of neuromuscular blockade |
|  | Intraoperative fluid management | Restrictive fluid therapy (defined as maintenance fluids at <2 ml/kg/h) or goal-oriented fluid therapy (stroke volume) |
|  | Multimodal analgesia | Use of more than two drugs or analgesia strategies (transversus abdominis plane (TAP) block or spinal anesthesia for minimally invasive surgery; thoracic epidural anesthesia for open surgery to reduce the use of opiates |
|  | Minimally invasive surgery | Patient submitted to laparoscopic, robotic or video-assisted surgery (conversions to open surgery included on an intention-to-treat basis) |
|  | No nasogastric tube | Nasogastric tube, if used, is removed at the end of surgery |
|  | No drain | No drain is placed in the abdominal cavity (pelvic drain allowed for pelvic surgery with low colorectal anastomosis) |
| Postoperative | Bladdder catheter | Urinary catheter removed on postoperative day after surgery (POD) 1 (up to POD 2 in case of pelvic surgery) |
|  | Early mobilization | Patient receives passive mobilization on POD 0, active mobilization on POD 1 |
|  | Early oral feeding | Patient receives liquid oral diet starting 6 hours after surgery and semisolid diet starting on POD 1 |
|  | Pre-discharge check | Patients are checked just before discharge at home concerning adequate oral intake, bowel function, adequate pain control, active mobilization, no clinical/serological evidence of any postoperative complication, full agreement to go home |

**Supplementary Table S4**

| **Covariate** | **SMD*** | **KS**** | **Reference Platform 1** |
| --- | --- | --- | --- |
| Sex | -0.01 | 0.01 | Platform2 |
| Age | 0.02 | 0.01 | Platform2 |
| American Society of Anesthesiologists Class | 0.01 | 0.01 | Platform2 |
| Body Mass Index | 0.01 | 0 | Platform2 |
| Diabetes | 0.05 | 0.02 | Platform2 |
| Intra- and/or postoperative blood transfusion(s) | 0.01 | 0 | Platform2 |
| Preoperative blood transfusion(s) | 0.05 | 0.01 | Platform2 |
| Chronic renal failure | 0.1 | 0.02 | Platform2 |
| Chronic liver disease | 0.08 | 0.01 | Platform2 |
| Center volume | 0.04 | 0.01 | Platform2 |
| Operation length | 0.06 | 0.03 | Platform2 |
| Overall items ERP adherence | 0.05 | 0.02 | Platform2 |
| Preoperative items ERP adherence | -0.25 | 0.12 | Platform2 |
| Intraoperative items ERP adherence | -0.07 | 0.03 | Platform2 |
| Postoperative items ERP adherence | 0.06 | 0.03 | Platform2 |
| Surgery for malignancy | 0.01 | 0.01 | Platform2 |
| Open Surgery | 0 | 0 | Platform2 |
| Type of resection | 0.04 | 0.02 | Platform2 |
| Intracorporeal anastomosis | -0.02 | 0.01 | Platform2 |
| Diameter of circular stapler | 0.12 | 0.06 | Platform2 |
| Center use of ≥ 2 circular stapler platforms | 0.01 | 0 | Platform2 |
| Sex | -0.03 | 0.01 | Platform3 |
| Age | -0.07 | 0.04 | Platform3 |
| American Society of Anesthesiologists Class | 0 | 0 | Platform3 |
| Body Mass Index | 0.08 | 0.04 | Platform3 |
| Diabetes | 0.17 | 0.05 | Platform3 |
| Intra- and/or postoperative blood transfusion(s) | 0.08 | 0.01 | Platform3 |
| Preoperative blood transfusion(s) | 0.16 | 0.03 | Platform3 |
| Chronic renal failure | 0.09 | 0.02 | Platform3 |
| Chronic liver disease | 0.08 | 0.01 | Platform3 |
| Center volume | 0.03 | 0.01 | Platform3 |
| Operation length | 0.13 | 0.06 | Platform3 |
| Overall items ERP adherence | 0.01 | 0.01 | Platform3 |
| Preoperative items ERP adherence | -0.3 | 0.14 | Platform3 |
| Intraoperative items ERP adherence | 0.01 | 0.01 | Platform3 |
| Postoperative items ERP adherence | 0 | 0 | Platform3 |
| Surgery for malignancy | -0.21 | 0.11 | Platform3 |
| Open Surgery | -0.05 | 0.01 | Platform3 |
| Type of resection | 0.11 | 0.05 | Platform3 |
| Intracorporeal anastomosis | -0.1 | 0.03 | Platform3 |
| Diameter of circular stapler | 0.58 | 0.27 | Platform3 |
| Center use of ≥ 2 circular stapler platforms | -0.03 | 0 | Platform3 |
| Sex | 0.04 | 0.02 | Platform4 |
| Age | 0.03 | 0.02 | Platform4 |
| American Society of Anesthesiologists Class | 0.09 | 0.04 | Platform4 |
| Body Mass Index | -0.13 | 0.06 | Platform4 |
| Diabetes | 0.09 | 0.03 | Platform4 |
| Intra- and/or postoperative blood transfusion(s) | -0.01 | 0 | Platform4 |
| Preoperative blood transfusion(s) | 0.11 | 0.02 | Platform4 |
| Chronic renal failure | 0.06 | 0.01 | Platform4 |
| Chronic liver disease | 0.06 | 0.01 | Platform4 |
| Center volume | 0.14 | 0.05 | Platform4 |
| Operation length | -0.01 | 0 | Platform4 |
| Overall items ERP adherence | 0 | 0 | Platform4 |
| Preoperative items ERP adherence | -0.26 | 0.12 | Platform4 |
| Intraoperative items ERP adherence | -0.17 | 0.08 | Platform4 |
| Postoperative items ERP adherence | 0.07 | 0.03 | Platform4 |
| Surgery for malignancy | -0.08 | 0.04 | Platform4 |
| Open Surgery | 0.02 | 0 | Platform4 |
| Type of resection | 0.1 | 0.04 | Platform4 |
| Intracorporeal anastomosis | -0.01 | 0 | Platform4 |
| Diameter of circular stapler | 0.09 | 0.04 | Platform4 |
| Center use of ≥ 2 circular stapler platforms | -0.05 | 0 | Platform4 |
| Sex | 0 | 0 | Platform5 |
| Age | 0 | 0 | Platform5 |
| American Society of Anesthesiologists Class | 0.13 | 0.06 | Platform5 |
| Body Mass Index | 0.01 | 0 | Platform5 |
| Diabetes | 0.06 | 0.02 | Platform5 |
| Intra- and/or postoperative blood transfusion(s) | 0 | 0 | Platform5 |
| Preoperative blood transfusion(s) | 0.09 | 0.02 | Platform5 |
| Chronic renal failure | 0.01 | 0 | Platform5 |
| Chronic liver disease | 0.09 | 0.01 | Platform5 |
| Center volume | 0.19 | 0.07 | Platform5 |
| Operation length | -0.04 | 0.02 | Platform5 |
| Overall items ERP adherence | 0.51 | 0.22 | Platform5 |
| Preoperative items ERP adherence | 0.17 | 0.08 | Platform5 |
| Intraoperative items ERP adherence | 0.14 | 0.07 | Platform5 |
| Postoperative items ERP adherence | 0.44 | 0.2 | Platform5 |
| Surgery for malignancy | 0.16 | 0.08 | Platform5 |
| Open Surgery | 0.04 | 0 | Platform5 |
| Type of resection | 0.07 | 0.03 | Platform5 |
| Intracorporeal anastomosis | -0.15 | 0.04 | Platform5 |
| Diameter of circular stapler | 0.87 | 0.41 | Platform5 |
| Center use of ≥ 2 circular stapler platforms | 0.12 | 0.01 | Platform5 |

*SMD: Standardized mean difference; **KS: Kolmogorov–Smirnov mean.

**References**

1.	Kaiser MJ, Bauer JM, Ramsch C, et al. Validation of the Mini Nutritional Assessment short-form (MNA-SF): a practical tool for identification of nutritional status. *J Nutr Health Aging*. 2009;13:782-788.

2.	Bujang MA, Sa'at N, Sidik TMITAB, et al. Sample Size Guidelines for Logistic Regression from Observational Studies with Large Population: Emphasis on the Accuracy Between Statistics and Parameters Based on Real Life Clinical Data. *Malays J Med Sci*. 2018;25:122-130.

3.	Peduzzi P, Concato J, Kemper E, et al. A simulation study of the number of events per variable in logistic regression analysis. *J Clin Epidemiol*. 1996;49:1373–1379.

4.	Catarci M, Ruffo G, Viola MG, et al. ERAS program adherence-institutionalization, major morbidity and anastomotic leakage after elective colorectal surgery: the iCral2 multicenter prospective study. *Surg Endosc*. 2022;36:3965-3984.

5.	Italian ColoRectal Anastomotic Leakage (iCral) study group. Patient-reported outcomes, return to intended oncological therapy and enhanced recovery pathways after colorectal surgery: a prospective multicenter observational investigation by the Italian ColoRectal Anastomotic Leakage (iCral 3) study group. *Ann Surg Open*. 2023;4:e267.

6.	Catarci M, Guadagni S, Masedu F, et al. Three versus two-rows circular staplers for left-sided colorectal anastomosis: a propensity score-matched analysis of the iCral 2 and 3 prospective cohorts. *Int J Surg*. 2023;109:2312–2323.

7.	McCaffrey DF, Beth Ann Griffin BA, Almirall D, et al. A Tutorial on Propensity Score Estimation for Multiple Treatments Using Generalized Boosted Models. *Stat Med*. 2013;32:3388–3414.

8.	VanderWeele TJ, Ding P. Sensitivity Analysis in Observational Research: Introducing the E-Value. *Ann Intern Med*. 2017;167:268-274.
